# The significance of CEACAM60, a carcinoembryonic antigen (CEA) homolog, as a tumor antigen in the porcine cancer model

**DOI:** 10.3389/fimmu.2026.1813834

**Published:** 2026-05-13

**Authors:** Jinsil Lee, Clara Cañas Crespo, Tim Hammacher, Andrei Lazar, Eva M Guenther, Marie Johne, Wiebke Weiß, Tung Huy Dau, Pavlo Maksimov, Krzysztof Flisikowski, Mykola Lyndin, Wolfgang Zimmermann, Hubertus Schleer, Robert Kammerer

**Affiliations:** 1 Institute of Immunology, Friedrich-Loeffler-Institut, Greifswald-Insel Riems, Germany; 2 Institute of Epidemiology, Friedrich-Loeffler-Institut, Greifswald-Insel Riems, Germany; 3 Surveillance Authority for Public Law Duties of the Medical Service East, Bundeswehr, Potsdam, Germany; 4 Chair for Infection Pathogenesis, Technical University Munich, Freising, Germany; 5 Institute of Anatomy, Medical Faculty, University of Duisburg-Essen, Essen, Germany; 6 Tumor Immunology Laboratory, LIFE Center, Department of Urology, Ludwig-Maximilians-University, Munich, Germany; 7 Genovac GmbH, Freiburg, Germany

**Keywords:** animal model, carcinoembryonic antigen (CEA), CEA-related cell adhesion molecule (CEACAM), colorectal cancer, pig

## Abstract

**Objective:**

Translational medicine remains one of the central challenges in modern biomedical research. A major bottleneck in this field is the limited availability of suitable animal models that reliably predict human disease outcomes. Large animal models, particularly pigs, are increasingly recognized as promising preclinical systems for translating fundamental immunological and oncological discoveries into clinically relevant applications.

**Methods:**

Given the pivotal role of the carcinoembryonic antigen (CEA) family, both as key regulators of immune responses (e.g., CEACAM1) and as tumor antigens (e.g., CEA) in humans, we performed a comprehensive characterization of the porcine CEA gene family. By generating monoclonal antibodies specific for individual porcine CEACAMs, we analyzed the expression of porcine CEACAMs in tissues and culture supernatants.

**Results:**

Our analyses identified the ortholog of CEACAM1 as well as a GPI-anchored CEACAM (CEACAM60), a homolog of human CEA. We demonstrated that CEACAM1 is expressed on various immune cell populations, consistent with patterns observed in humans. Furthermore, we found that CEACAM60 is selectively expressed at the apical membrane of enterocytes in the large intestine, mirroring the expression topology described for human CEA. Strict apical localization in healthy epithelial tissues results in the release of CEACAM60 into the intestinal lumen, thereby preventing its presence in systemic body fluids. In contrast, in malignant tissues, loss of cellular differentiation and disruption of apical-basal polarity abolish this compartmentalization, providing the essential prerequisite for the occurrence of CEACAM60 in serum under neoplastic conditions. Based on these findings, we developed a detection assay for CEACAM60 in fluids, which could serve as a basis for dynamic monitoring of tumor progression in animals in the future.

**Conclusion:**

Considering the central roles of CEACAM1 and CEA in tumor immunotherapy and tumor surveillance in humans, porcine models with an increased predisposition for colorectal neoplasia may provide a valuable translational platform for the development and refinement of next-generation colorectal cancer immunotherapies.

## 1 Introduction

Murine models, especially when they were genetically modified, have proven to be very useful to understand the mechanism of certain human diseases (1). However, in certain circumstances, murine models are not sufficient to optimize new diagnostic and therapeutic approaches (2). Frequently, the size and anatomy of the model animal are critical constrains, but sometimes also the physiology of humans exclude mice or rodents as adequate model organisms. Thus, there is a clear need for alternative animal models. The development of novel techniques for genetic manipulation of large animals has opened the door to generate more relevant animal models (3, 4). One of such model species is the swine, which, due to its anatomy and physiology, is particularly suitable for studies on diabetes, heart diseases and certain types of cancer (5–7). Indeed, a new promising pig model of colorectal cancer was recently described (8, 9). This model is based on the genetic introduction of a mutation into the *adenomatous polyposis coli* (APC) gene that is orthologous to that responsible for human familial adenomatous polyposis (FAP). One limitation of this model is that, so far, progression of the polyps to invasive carcinomas has not been reported (10). In this model colonoscopy a very important diagnostic procedure can be easily performed. However, to optimize therapeutic strategies simpler methods of monitoring neoplasms are desirable. Although different biomarkers can be used, in humans the determination of the serum tumor marker carcinoembryonic antigen (CEA) is still relevant and can even have prognostic significance when combined with CA19–9 in colorectal cancer (11). CEA, also called CEACAM5, is a member of the CEA family described more than half a century ago by Gold and Freedman and independently by von Kleist and Burtin (12, 13). The CEA family is a member of the immunoglobulin superfamily and comprises five conserved genes for which orthologues can be identified in most mammalian species studied so far (14). One of these genes, *CEACAM1*, has given rise to a number of species-specific paralogs, classified into two subgroups called the CEA-related cell adhesion molecule (CEACAMs) genes and the pregnancy-specific glycoprotein (PSGs) genes. PSGs exist only in certain mammals and are defined by their trophoblast-specific expression (15, 16). The CEACAM1 paralogs can either be secreted, glycosylphosphatidylinositol (GPI)-linked, or transmembrane proteins (17). The transmembrane-anchored CEACAMs have short or long cytoplasmic tails which often contain inhibitory or activating motifs suggesting that many CEACAMs are important signaling molecules that regulate cell-cell-communication (17). The GPI linked CEACAMs are also involved in cell signaling (18, 19). Remarkably, so far, no GPI-linked CEACAMs were described in non-primate mammals, indicating that the GPI linkage of CEACAMs is primate-specific. Interestingly, the generation of GPI-linked CEACAMs independently evolved in both Old World and New World monkeys through convergent evolution, suggesting a selective pressure favoring the emergence of these molecules in primates (20). More recently, a member of the CEACAM protein family was characterized in zebrafish and predicted to be GPI-anchored. This CEACAM family member was found to be specifically expressed in H^+^-ATPase-rich (HR) cells, a specialized type of ionocytes located in the fish epidermis and gills (21). The GPI anchor is a glycolipid structure that is post translationally attached to the C-terminus of many eukaryotic proteins. This modification anchors the protein in the outer leaflet of the cell membrane. Although the functional significance of the GPI anchor is not completely elucidated, cumulative evidence suggests functional roles of the GPI anchor in lipid raft partitioning, signal transduction and apical membrane targeting (22). In addition, GPI-anchored membrane molecules can be cleaved from the cell surface by phosphatidylinositol phospholipase C (PI-PLC) and D (PI-PLD) (22). Therefore, GPI-anchored proteins are secreted molecules that are retained at the cell surface for a certain time period. Roughly 10–20% of all eukaryotic membrane proteins that enter the secretory pathway are GPI-anchored (23). For the usage of CEA as a target antigen in tumor therapies and in the same time as a tumor marker, the GPI linkage is very important, because it guaranties the expression of CEA at the tumor cell surface but also allows the release from the surface and its appearance in the serum where it is easily accessible in clinical settings for tumor surveillance. Previously, we have analyzed the CEA gene family in dogs in order to identify new biomarkers for canine mammary cancer (24). Here we describe the CEA gene family in pigs and, in particular, assess the potential of CEACAM60, the first GPI−anchored CEACAM identified in a non−primate mammal, as a potential biomarker and/or therapeutic target for anticancer strategies in the pig model.

## 2 Results

### 2.1 Identification of the CEA gene family in the porcine genome

In order to identify the members of the CEA gene family in pigs, we searched the Sscrofa10.2 genome (breed Duroc), whole-genome shotgun (WSG) databases (breeds: Duroc, Ellegaard Göttingen minipig, Tibetan, Wuzhishan), and expressed sequence tags (EST) databases containing various breeds and crossbreeds with sequences of known CEACAMs in other species, including human, mouse, rat, dog and cattle. Orthologous genes to the conserved CEA family members *CEACAM1*, *CEACAM16*, *CEACAM18*, *CEACAM19*, and *CEACAM20* were found. Interestingly, no sequences from *CEACAM20* were detected in the Sscrofa10.2 genome assembly, however the complete sequence of *CEACAM20* is now present in the Sscrofa11.1 assembly. The CEA gene family locus is located on chromosome 6 with the following gene identifiers *CEACAM1* (annotated), *CEACAM16* (annotated), *CEACAM18* (annotated), *CEACAM19* (annotated) and *CEACAM20* (LOC100627471).

**Figure 1:**
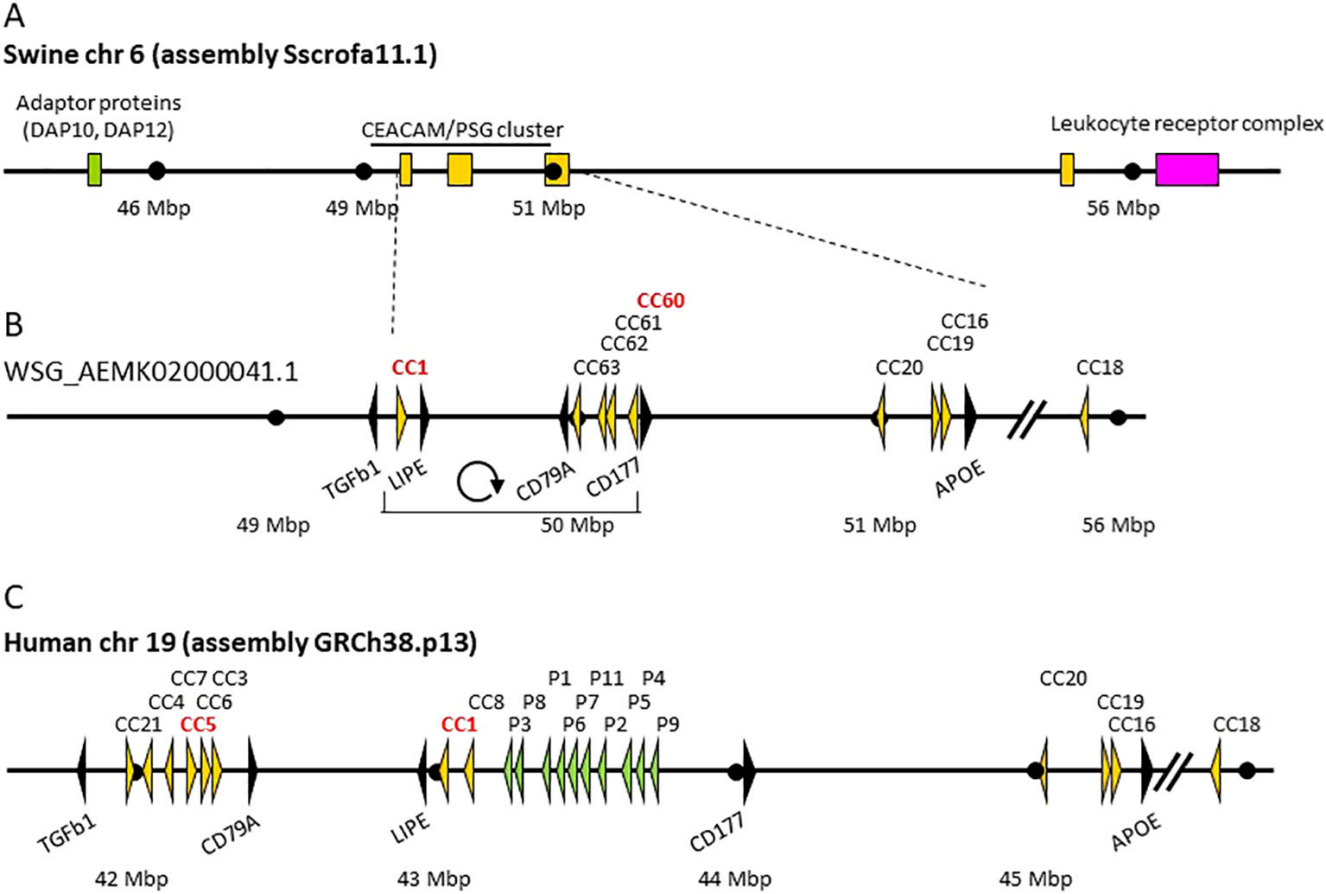
Genomic arrangement of the porcine extended leukocyte receptor complex and comparison of the CEACAM/PSG locus in swine and humans. **(A)** Genomic organization of swine CEA gene family locus on chromosome (chr) 6 according to the assembly version Sscrofa11.1. **(B)** CEACAM locus in the swine genome (the sequence is derived from the whole genome shotgun sequence AEMK02000041.1). Please note that the order of non-CEACAM genes between the flanking genes TGFB1 and CD177 differs between humans and swine. An inversion of the genomic region encompassing CD79A, LIPE and flanking CEACAM genes can be seen. **(C)** CEACAM/PSG locus on human chromosome 19 according to the assembly GRCh38.p13. Arrowheads represent genes with their transcriptional orientation. CEACAM (CC) genes are shown in yellow, PSG genes (P) in green and marker genes in black. The funder gene of the CEA gene family CEACAM1, human CEA gene (CEACAM5) and porcine CEACAM60 are highlighted in red. The scale indicated by dots is 1 Mbp unless interrupted by slanted lines. Note the inversion between *TGFb1* and *CD177* of the swine genome compared to the human genome.

In mammals, duplications of the *CEACAM1* gene give rise to the expansion of the CEA gene family. Therefore, we next searched databases for CEACAM1-related CEACAMs using the nucleotide sequence of the swine CEACAM1 N domain exon encoding the N-terminal immunoglobulin variable (IgV)-like domain. We identified four additional N domain exon sequences, none of them belonged to an already annotated gene. Thus, we performed blast searches with other exon sequences known from other CEA gene families and identified four putative genes which we named *CEACAM60* to *CEACAM63*. Two of these genes were found in the Sscrofa10.2 genome (*CEACAM60* and *CEACAM61*). All four genes were present in the WSG database for the Tibetan, Wuzhishan and Ellegaard Göttingen minipig breeds. In the Sscrofa11.1 assembly these genes are merged and annotated with the identifier (LOC102158679) located between the gene coding for CD79A and the gene coding for CD177 (**Figure 1**).

### 2.2 Structure and splice variants of the porcine CEA gene family members

**Figure 2:**
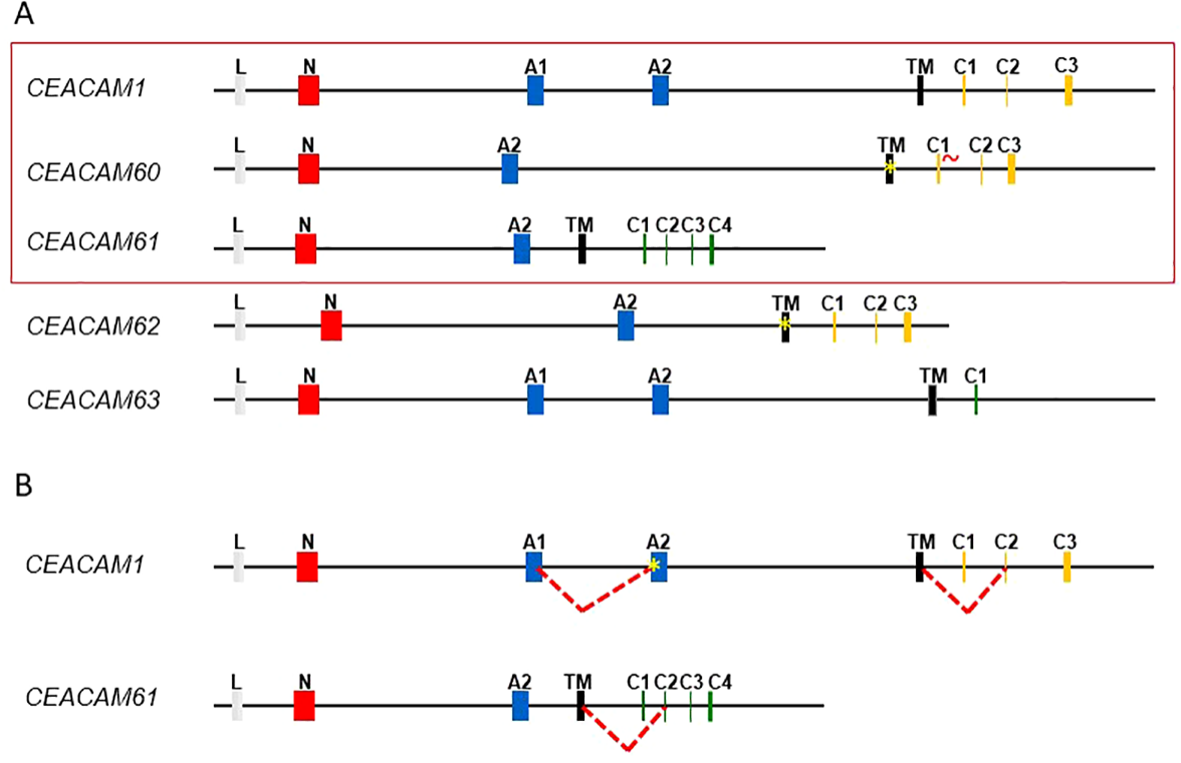
Exon structure of CEACAMs in the pig genome and transcript variants. **(A)** The exon types are indicated by differently colored boxes. Leader sequences (L) are shown as light gray, IgV-like domain exons (N) as red, IgC-like domain exons (A1 = A1-type and A2 = A2-type) as blue and transmembrane domain exons (TM) as black boxes. The exons encoding the cytoplasmic domain (C1-C4) with an ITIM or an ITAM motif are shown in yellow and green, respectively. The red wavy line next to C1 in CEACAM60 indicates that the splice donor site is degenerated. The presence of a stop codon is indicated by an asterisk. Cloned sequences are indicated by a red frame. **(B)** Alternative splice events are indicated by the red dashed line. New stop codons induced by frame shifts are indicated with a yellow asterisk.

We used the exon sequences identified from the blast searches to predict the structure of the porcine *CEACAM1*-related genes (**Figure 2A**). In most mammalian species the *CEACAM1* gene is composed of nine exons encoding the following domains: leader, N, immunoglobulin constant (IgC)-like domains A1, B, A2, transmembrane domain TM, cytoplasmic domains C1, C2, C3. The three cytoplasmic exons encode either one immunoreceptor tyrosine-based inhibitory motif (ITIM) and one immunoreceptor tyrosine-based switch motif (ITSM) or two ITIM motifs. The *CEACAM1* gene in pigs lacks a B domain exon similar to bovine *CEACAM1* [Kammerer et al., 2004], indicating that the B domain exon in the *CEACAM1* gene was lost before evolutionary divergence of pigs and cattle. CEACAM60 is composed of one N domain and one A domain. *CEACAM60* has a stop codon in the TM exon and degenerated splice donor sites in the first cytoplasmic exon, homologous to the cytoplasmic exon 1 found in the ITIM/ITSM-encoding CEACAM1. CEACAM61 comprises an N and an A domain, followed by a transmembrane region and a cytoplasmic domain containing an immunoreceptor tyrosine-based activation motif (ITAM), similar to that found in human CEACAM3. CEACAM62 is composed of an N domain and an A2 domain, followed by a TM that contains a stop codon near its C-terminal end. This arrangement allows the domain to fully span the membrane, leaving three amino acids exposed to the cytoplasmic space. CEACAM63 has two A domains. Interestingly, *CEACAM63* has also a sequence similar to a B domain exon next to the A1 exon. However, the splice acceptor site of the putative B domain exon is mutated. Similar to CEACAM62, CEACAM63 possesses a TM exon followed by a very short cytoplasmic tail consisting of only a few amino acids.

Based on these predictions, we designed specific primers (**Table 1**) to amplify full-length cDNA by RT-PCR from various porcine tissues. CEACAM1, CEACAM60, and CEACAM61 cDNAs were successfully amplified, and the predicted gene products were confirmed by Sanger sequencing. In contrast, we were not able to amplify enough cDNA of CEACAM62 and CEACAM63 from any analyzed tissue to allow cloning and sequencing. Consistently, no EST sequences corresponding to these genes were found in the EST database at NCBI. In contrast multiple EST sequences corresponding to each of the genes CEACAM1, CEACAM60 and CEACAM61 were present in the database. However, more recently we identified several mRNA sequences of CEACAM62 and CEACAM63 in the Transcriptome shotgun assembly (TSA) database at NCBI, indicating that these genes are expressed in certain tissues. The cloning of CEACAM1 and CEACAM61 cDNAs revealed several splice variants (**Figure 2B**). Similar to observations in other mammalian species, one porcine CEACAM1 splice variant lacked the first cytoplasmic exon, resulting in a frameshift and a markedly shortened cytoplasmic tail lacking ITIM or ITSM motifs. Furthermore, an alternative splicing event using a different splice acceptor site of exon 4 introduces a frameshift and a premature stop codon, producing a secreted form of CEACAM1 composed of an N and an A1 domain. For CEACAM61, an alternative splice variant was identified in which exclusion of the first cytoplasmic exon (53 bp in length) generates a shorter cytoplasmic tail lacking the ITAM-like motif (**Figure 2B**). For the complete nucleotide sequences of identified porcine CEACAMs see **Supplementary Data 1**.

**Table 1:** Gene-specific oligonucleotides for expression analyses and cDNA cloning of swine CEA gene family members.

Gene	oligonucleotide name	Oligonucleotide sequence	Location of primers (exon)	PCR product size (bp)
CEACAM1	Ssc_CC1_fo_L Ssc_CC1_re_N	For: 5’_CCCACAGAGGGCATGTCC Rev: 5’_GGTATATGCTTGAGTGTCTATTCTG	L N	245
CEACAM60	Ssc_CC60_fo_L Ssc_CC60_re_N	For: 5’_CCACGGAGGGCGCATCT Rev: 5’_GCTTGAATGTCTACTCTATATAA	L N	240
CEACAM61	Ssc_CC61_fo_L Ssc_CC61_re_N	For: 5’_GCCACAGAGGGCATGTCC Rev: 5’_AGTTACTTGACGGTCTACTTCA	L N	245
CEACAM62	Ssc_CC62_fo_L Ssc_CC62_re_N	For: 5’_CCACGGAGGGCGCATCT Rev: 5’_GTTAGTTGAAAGTCTACTGCATG	L N	243
CEACAM63	Ssc_CC63_fo_L Ssc_CC63_re_N	For: 5’_CCACGGAGGGCGCATCT Rev: 5’_AGATTGTCTCTTGACTGCTCTG	L N	281
CEACAM1	Ssc_NotI_CC1_fo Ssc_CC1_XbaI_re	For: 5’_GTCAGTGCGGCCGCTCTCTGACAGGG AGGGACAC Rev: 5’_GTCAGTTCTAGAGAGCAGGACTGGTT GCATTAC	5’UTR 3’UTR	1347
CEACAM60	Ssc_CC60_HindIII_fo Ssc_CC60_XbaI_re	For: 5’_GTCAGTAAGCTTCAGATCACTATTGA ATCAGTGCCC Rev: 5’_GTCAGTTCTAGAGAGCAGGACTGGTT GCATTAC	5’UTR 3’UTR	932
CEACAM61	Ssc_CC61_5’_fo Ssc_CC61_3’_re	For: 5’_TCTCACAGGGAGGGACACA Rev: 5’_GGAGAAGTTCCTGGCAGTCA	5’UTR 3’UTR	1071
GAPDH	Ssc_GAPDH_fo Ssc_GAPDH_re	For: 5**’**_CCTTCATTGACCTCAACTACAT Rev: 5**’**_CCAAAGTTGTCATGGATGACC		400

### 2.3 Phylogeny of CEACAM1 paralogs

**Figure 3:**
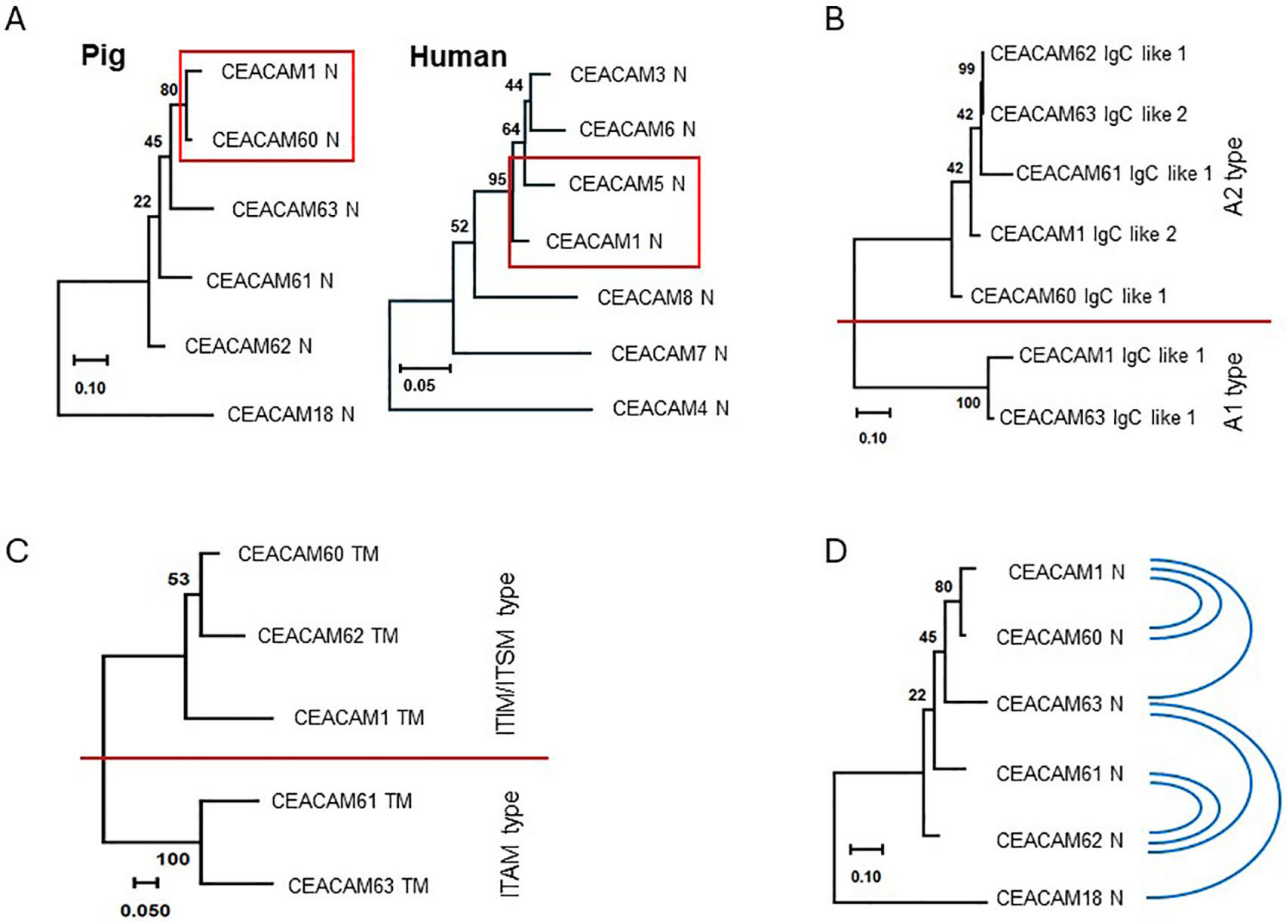
Phylogeny of porcine CEACAM genes. **(A)** The phylogenetic tree was constructed using nucleotide sequences of N domain exons of CEACAM1-like CEACAMs in the pig and in humans. Note the close similarity between pig CEACAM60 and CEACAM1 and between human CEACAM5 and CEACAM1 sequences (red frames). **(B)** Phylogenetic tree of A domain exon nucleotide sequences is shown. Two types of A domains exist one is similar to CEACAM1 A1 and the other to CEACAM1 A2. In pigs the A domains belong mainly to the A2 type, only the A domain of CEACAM1 and CEACAM63 correspond to the A1 type (separated by the red line). **(C)** The similarity of the transmembrane exon sequence of CEACAM60 (TM) and the transmembrane exon sequence CEACAM1 (TM) indicates that the GPI linkage is derived from a modified transmembrane domain of the ITIM type usually found in CEACAM1 and not from the ITAM type found in CEACAM61 or human CEACAM3. **(D)** Gene conversion events between N domain exons of porcine CEACAMs are indicated using blue lines (see also **Table 2**). Note the two events between CEACAM1 and CEACAM60. Numbers at the nodes indicate the percentage of trees in which the associated taxa clustered together. The scale at the bottom of the tree indicates the number of substitutions per site.

To elucidate the phylogenetic relationships among the CEACAM1 paralogous genes in pigs, we performed phylogenetic analyses. We first compared the N domains, which are primarily responsible for ligand interactions, to assess sequence similarities. The results revealed that the N domain nucleotide sequence of CEACAM1 is most closely related to that of CEACAM60. This relationship mirrors the situation in humans, where the N domain of CEACAM1 shows high similarity to that of CEA/CEACAM5 (**Figure 3A**). Next, we aimed to characterize the IgC-like A domains in more detail and to classify them as either CEACAM1 A1 or A2 type. As shown in **Figure 3B**, the IgC-like domains are predominantly of the A2 type. Only CEACAM1 and CEACAM63 possess A1 domains, whereas all remaining A domains belong to the A2 type. In most mammalian CEA gene families, two distinct types of TM are present: one originates from the CEACAM1 TM and is linked to inhibitory signaling motifs in the cytoplasmic region, whereas the other, of yet unclear origin, is associated with activating motifs in the corresponding cytoplasmic domains. Both types are frequently found within the same species, forming so-called paired receptors. **Figure 3C** shows that the TM of CEACAM60 and CEACAM62 are derived from CEACAM1, whereas those of CEACAM61 and CEACAM63 are of the activating type. However, neither CEACAM60 and CEACAM62 nor CEACAM63 possess the corresponding cytoplasmic signaling motifs (**Figure 2A**). Gene conversion is a dominant mechanism to keep the sequence of the ligand-binding domains (N domains) of CEACAM genes similar to each other (25–27). Therefore, we analyzed gene conversion events in the N domain exons of porcine *CEACAMs*. Seven different events were detected (**Figure 3D**; **Table 2**). Two were found between *CEACAM1* and *CEACAM60* and two between *CEACAM61* and *CEACAM62*. *CEACAM63* was a partner for a single gene conversion for *CEACAM1*, *CEACAM62* and *CEACAM18*. The results suggest that gene conversion serves as an important mechanism for homogenizing the N domains of porcine CEACAMs.

**Table 2:** Gene conversions (global inner fragments (bold) and pairwise inner fragments) detected with GENECONV.

#	Sequences	Sim P value	BC KA P value	Begin	End	length
1	**CEACAM61;CEACAM62**	**0.0159**	**0.02575**	**40**	**109**	**70**
#	sequences	Sim P value	KA P value	Begin	End	length
1	CEACAM1;CEACAM60	0.0152	0.04669	257	389	133
2	CEACAM1;CEACAM60	0.0439	0.09505	84	176	93
3	CEACAM1;CEACAM63	0.0300	0.04411	31	69	39
4	CEACAM61;CEACAM62	0.0000	0.00021	40	109	70
5	CEACAM61;CEACAM62	0.0150	0.02670	212	279	68
6	CEACAM62;CEACAM63	0.0450	0.06543	12	47	36
7	CEACAM62;CEACAM18	0.0165	0.02003	159	167	6

Gene conversion within the N domain exon (exon 2); Sim P value, Simulated P-values based on 10,000 permutations; BC KA P value, Bonferroni-corrected KA (BLAST-like) P-values; KA P values are not Bonferroni-corrected.

Bold values indicate global inner fragments.

### 2.4 CEACAM60 is preferentially expressed in the large intestine

**Figure 4:**
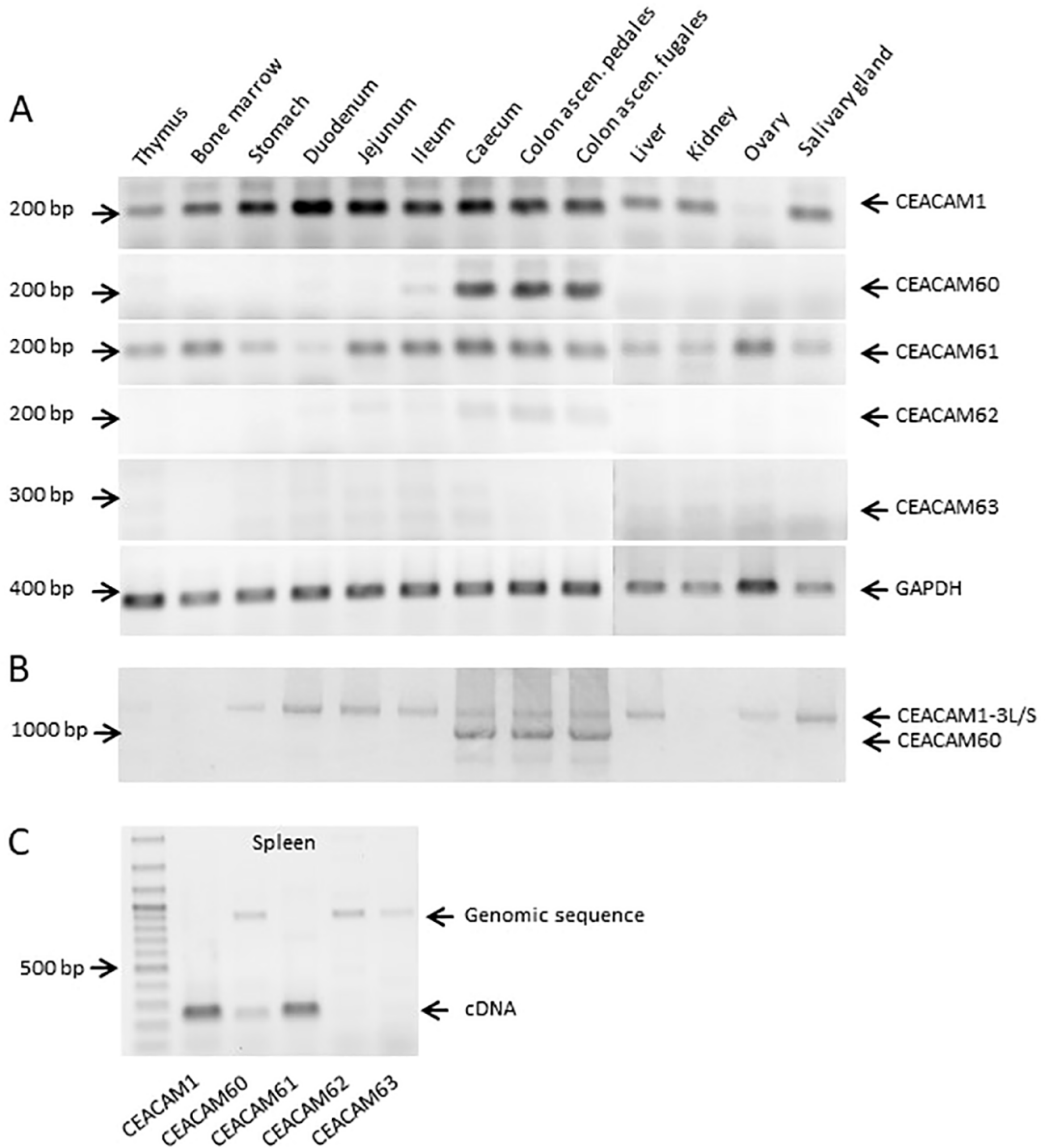
CEACAM60 is preferentially expressed in the large intestine. **(A)** RNA was isolated from various tissues and RT-PCR was performed as described in “Material and Methods”. mRNA coding for CECAM1 and CEACAM61 was found in several tissues. mRNA coding for CEACAM60 could be detected in caecum and colon and to a much lesser extend in the ileum. Some expression of CEACAM62 was also seen in the intestine. A significant expression of CEACAM63 was not found. GAPDH was used as a control for the integrity of the isolated mRNA. **(B)** The expression of CEACAM60 in the large intestine was further verified by the amplification of full-length CEACAM1 and CEACAM60 using a primer pair that amplifies both gene products. **(C)** The specificity of the primers used was tested using RNA isolated from spleen. These preparations contain in addition to the RNA also some genomic DNA. Specific products from all genes were amplified either from cDNA or from the genomic DNA or both. Marker, 100 bp ladder.

Next, we analyzed the expression of the various porcine CEACAMs in different tissues using semi−quantitative RT−PCR. In addition to the full−length primers described above, we designed gene−specific primers (**Table 1**) to amplify sequences spanning the leader region and the N domain−encoding exon. Using these primers, we screened CEACAM expression across multiple tissues (**Figure 4A**). *CEACAM1* and *CEACAM61* showed broad expression, indicating that these genes are either ubiquitously expressed or associated with cell types widely distributed among tissues. By contrast, *CEACAM60* expression was restricted to the large intestine, specifically the caecum and colon. This expression pattern closely resembles that of human *CEA*/*CEACAM5* in the digestive tract. Very weak signals for *CEACAM62* were detected only in tissues of the large intestine, while *CEACAM63* expression was not observed in any tissue analyzed (**Figure 4A**). Amplification of full−length CEACAM60 (**Figure 4B**) provided no indication of alternative splice isoforms. In spleen tissue, two main products were amplified, one corresponding to CEACAM1 and the other to CEACAM61 (**Figure 4C**). A faint signal was also detected for CEACAM60, whereas CEACAM62 and CEACAM63 were absent. Amplifications from genomic DNA confirmed primer specificity (**Figure 4C**), indicating that the lack of amplification from cDNA reflects true absence of mRNA expression for these genes.

### 2.5 CEACAM60 is linked to the cell membrane by a GPI linker

**Figure 5:**
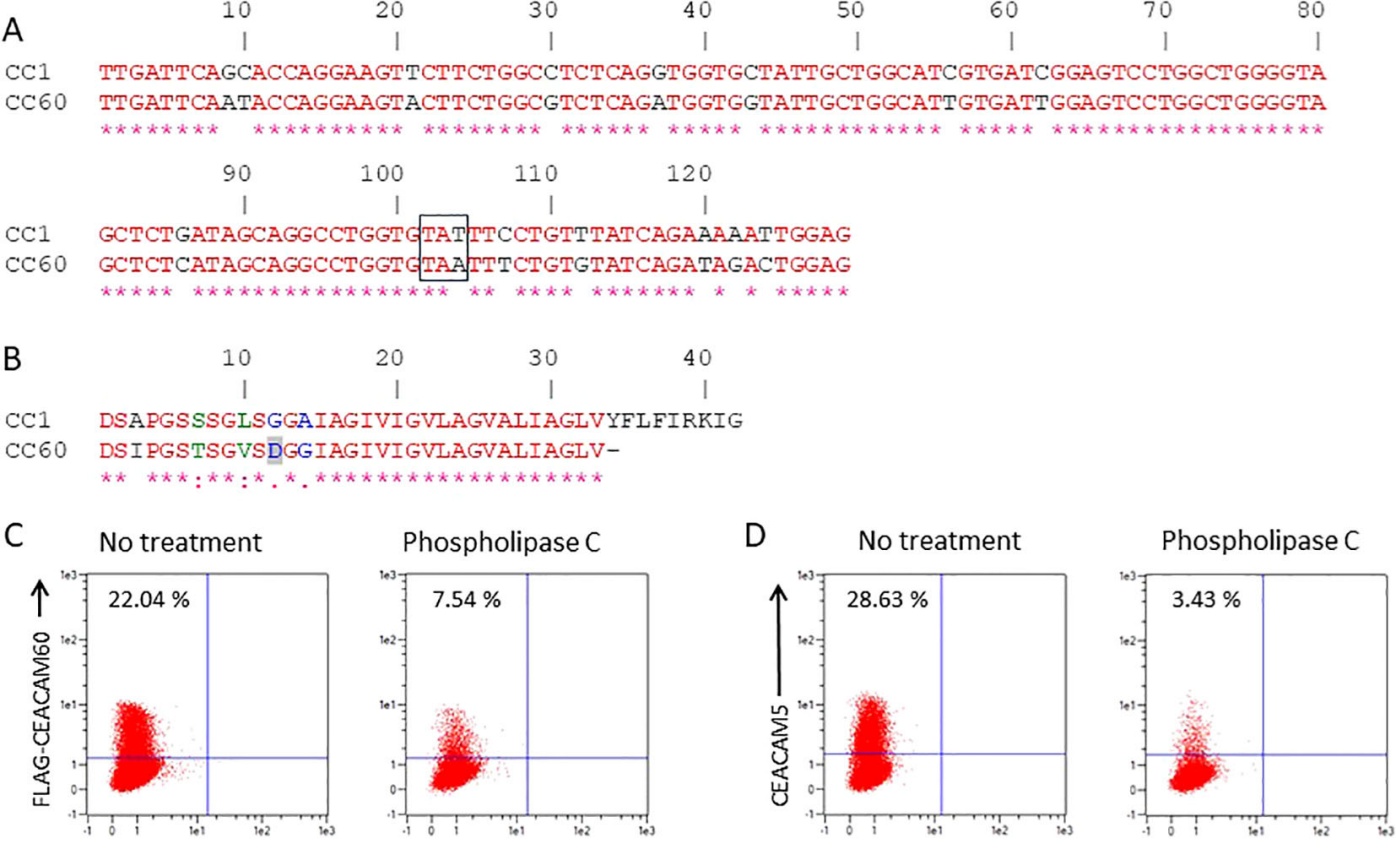
CEACAM60 is GPI-anchored. **(A)** The membrane domain sequence of CEACAM60 is closely related to the transmembrane domain sequence of CEACAM1. Identical nucleotides are shown in red. In CEACAM60 a single nucleotide substitution T to A created a TAA stop codon (marked with a black frame). **(B)** Comparison of the amino acid sequence from the transmembrane domain of CEACAM1 and CEACAM60. The degree of conservation of the sequences is shown at the bottom of the graph [* = identical (red),: = conservative changes (green),. = less stringent conservative changes (blue)]. The ω site predicted is highlighted in gray. **(C, D)** Cos7/L cells were transfected with N-terminally Flag-tagged CEACAM60 cDNA or human CEA/CEACAM5 cDNA and treated with or without phosphatidylinositol-specific phospholipase C (PI-PLC) for 50 min at 37 °C. The amount of CEACAM60 and CEA on the cell surface was quantified by flow cytometry using specific mAbs. A representative result from three independent experiments is shown.

The transmembrane domain of CEACAM60 contains an internal stop codon, resulting in a nonfunctional transmembrane region (**Figure 5**). In the human CEA family, such incomplete transmembrane domains are replaced by a GPI anchor, as described for CEACAM5, CEACAM6, CEACAM7 and CEACAM8. We therefore speculated that CEACAM60 could be also subject of posttranslational modification and that the transmembrane domain might be replaced by a GPI anchor. We therefore analyzed the CEACAM60 sequence using various GPI prediction programs such as PredGPI, big-PI-Predictor and GPI-SOM. All three programs predicted that CEACAM60 is GPI-linked with a moderate probability score (data not shown). The PredGPI and the big-PI-Predictor determined the ω-site at amino acid sequence position 246 and 247/246, respectively (**Figure 5B**). Next, we tested whether CEACAM60 could be released from the cell surface by phospholipase C treatment. To this end, we transfected Cos7L cells with either Flag-tagged CEACAM60 or human CEA/CEACAM5 and analyzed their surface expression by flow cytometry. As shown in **Figures 5C, D** phospholipase C (PLC) treatment released both CEACAM60 and CEA from the cell surface in a comparable manner. The GPI anchoring of CEA, which is known to be sensitive to PLC, served as a positive control in this experiment.

### 2.6 Reactivity of monoclonal antibodies (mAbs) directed against porcine CEACAMs

**Figure 6:**
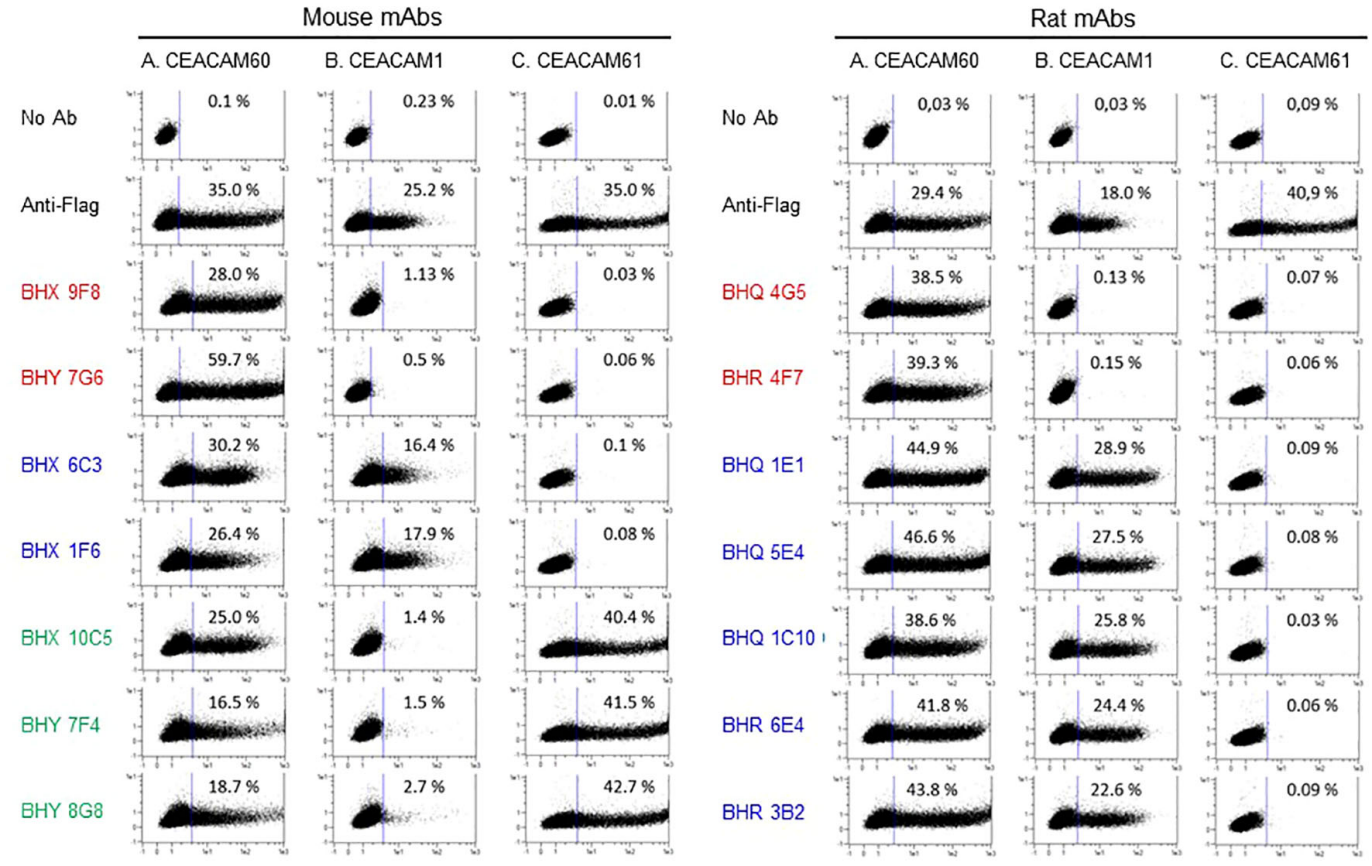
Mouse and rat mAb detect porcine CEACAMs. mAb against CEACAM60 were used to stain cells that were transfected with either Flag-tag-labeled CEACAM60 (columns A), CEACAM1 (columns B) or CEACAM61 (columns C). mAbs from mice (left panel) and mAbs from rats (right panel) were tested for binding to CEACAM60, CEACAM1 and CEACAM61. All antibodies recognized CEACAM60. mAbs specific for CEACAM60 are shown in red (clone name), mAbs cross-reacting with CEACAM1 were shown in blue and mAbs cross-reacting with CEACAM61 are shown in green. The percentage of positively stained cells is indicated in the dot blot.

As CEACAM60 represents the first GPI-anchored CEACAM identified in a non-primate mammal, we sought to generate monoclonal antibodies against this protein. To this end, the CEACAM60 sequence was provided to Genovac GmbH (Freiburg, Germany), which employed genetic immunization to produce CEACAM60-specific hybridomas in mice and rats. The reactivity of these hybridomas was subsequently characterized by flow cytometry using Flag-tagged CEACAM1-, CEACAM60-, and CEACAM61-transfected cells (**Figure 6**). Two mouse mAbs (BHX 9F8, BHY 7G6) and two rat mAbs (BHQ 4G5, BHR 4F7) were found to be specific for CEACAM60, showing no cross-reactivity with CEACAM1 or CEACAM61. None of the mAbs bound to all three tested CEACAMs. All cross-reacting rat mAbs bound to CEACAM60 and CEACAM1. No cross-reactivity of rat mAbs with CEACAM61 was observed (**Figure 6**). Two mouse mAbs (BHX 6C3, BHX 1F6) recognized CEACAM60 and CEACAM1 and three mouse mAbs (BHX 10C5, BHY 7F4, BHY 8G8) recognized CEACAM60 and CEACAM61. Finally, we confirmed reactivity of the mAbs with non-Flag-tagged CEACAMs (data not shown).

### 2.7 Expression of porcine CEACAMs by peripheral blood leucocytes

**Figure 7:**
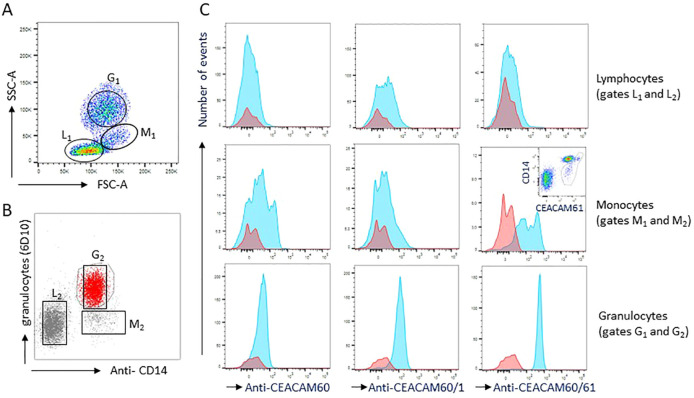
Expression of CEACAMs on porcine leucocytes. Flow cytometry of peripheral blood leucocytes was performed using anti-CEACAM60 (BHX 9F8), anti-CEACAM60/CEACAM1 (BHQ 1E1), and anti-CEACAM60/CEACAM61 (BHX 10C5) mAbs. Lymphocytes, monocytes and granulocytes were identified according to the forward and side scatter and the staining pattern of the mAbs 6D10 (granulocytes) and anti-CD14 mAb MIL2 (granulocytes and monocytes) **(A, B)**. **(C)** Overlay of histograms from control (red) and specific staining (blue). The histogram in the middle of the right panel has an insert demonstrating the higher expression of CEACAM61 by CD14^high^ monocytes. Monocytes are marked by a gray outline. L_1_ and L_2_, lymphocyte gates; M_1_ and M_2_, monocyte gates; G_1_ and G_2_, granulocyte gates.

We next used the generated mAbs to analyze the expression of CEACAMs on porcine peripheral blood leucocytes. Based on their forward and side scatter (FCS/SSC) characteristics, porcine leucocytes can be separated into lymphocytes, monocytes, and granulocytes (**Figure 7A**). Unlike in humans, where CD14 is restricted to monocytes, both monocytes and granulocytes express CD14 in pigs. Therefore, we performed double staining of peripheral leucocytes with anti-CD14 mAb and the granulocyte-specific mAb 6D10. This combination of markers enabled discrimination between monocytes and granulocytes (**Figure 7B**). CEACAM expression of individual leukocyte populations was determined only for cells identified as the respective subtypes in both FSC/SSC gating and the double-staining analysis. No significant CEACAM60 expression (mAb, clone BHX 9F8) was detected on porcine peripheral leucocytes. However, CEACAM1 (mAbs, clone BHQ 1E1) was detected on granulocytes and CEACAM61 (mAbs, clone BHX 10C5) was detected on both granulocytes and monocytes (**Figure 7C**).

### 2.8 CEACAM60 is expressed at the apical surface of enterocytes

**Figure 8:**
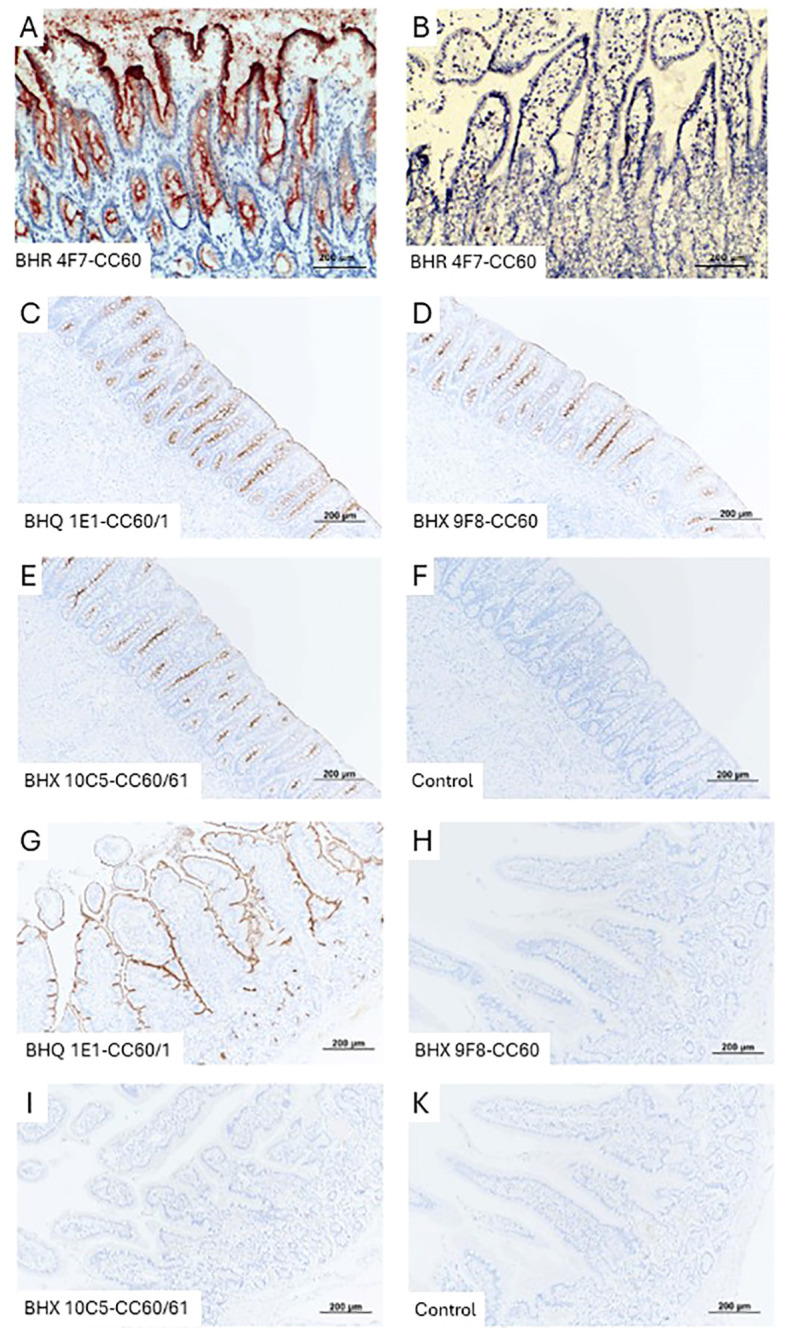
CEACAM60 is expressed on the apical surface of epithelial cells in the large intestine. Cryosections from colon **(A)** and jejunum **(B)** from the same animal were stained with the ABC method. The brown color indicates positive reaction. The staining indicates cell surface expression of CEACAM60 and the presence of CEACAM60 in the glycocalyx. The rat CEACAM60-specific mAb (BHR 4F7-CC60) was used. **(C–F)** paraffin sections from colon tissues and from jejunum **(G–K)**. The colon shows positive staining at the cell surface along the entire crypt with the anti-CEACAM60 (BHX 9F8-CC60) mAb. A similar staining occurred with the dual-specific anti-CEACAM60/CEACAM1 (BHQ 1E1-CC60/1) mAb and the anti-CEACAM60/CEACAM61 (BHX 10C5-CC60/61) mAb. The jejunum shows a positive staining at the cell surface on the villi and in the crypts with the BHQ 1E1-CC60/1 mAb. No staining is observed using the BHX 9F8-CC60 mAb, the BHX 10C5-CC60/61 mAb or the isotype control mAb.

To further investigate CEACAM60 expression in the intestine, we performed immunohistochemical analyses on both cryosections (**Figures 8A, B**) and paraffin−embedded sections (**Figures 8C–K**). In cryosections, CEACAM60 expression was detected in colonic enterocytes (**Figure 8A**) but not in enterocytes of the small intestine (**Figure 8B**). Staining of paraffin sections revealed pronounced CEACAM60 signal at the apical membrane of colonic enterocytes (**Figures 8C–F**). In contrast, small intestinal tissue (**Figures 8G–K**), showed a positive staining only with mAb BHQ 1E1, which recognizes CEACAM1, whereas no staining was obtained with the CEACAM60−specific mAb BHX 9F8. These findings indicate that CEACAM1 is expressed by enterocytes in both the villi and crypts of the small intestine, while CEACAM60 is expressed with comparable intensity by enterocytes throughout the crypts of the colon.

### 2.9 CEACAM60 is present in colon polyps

**Figure 9:**
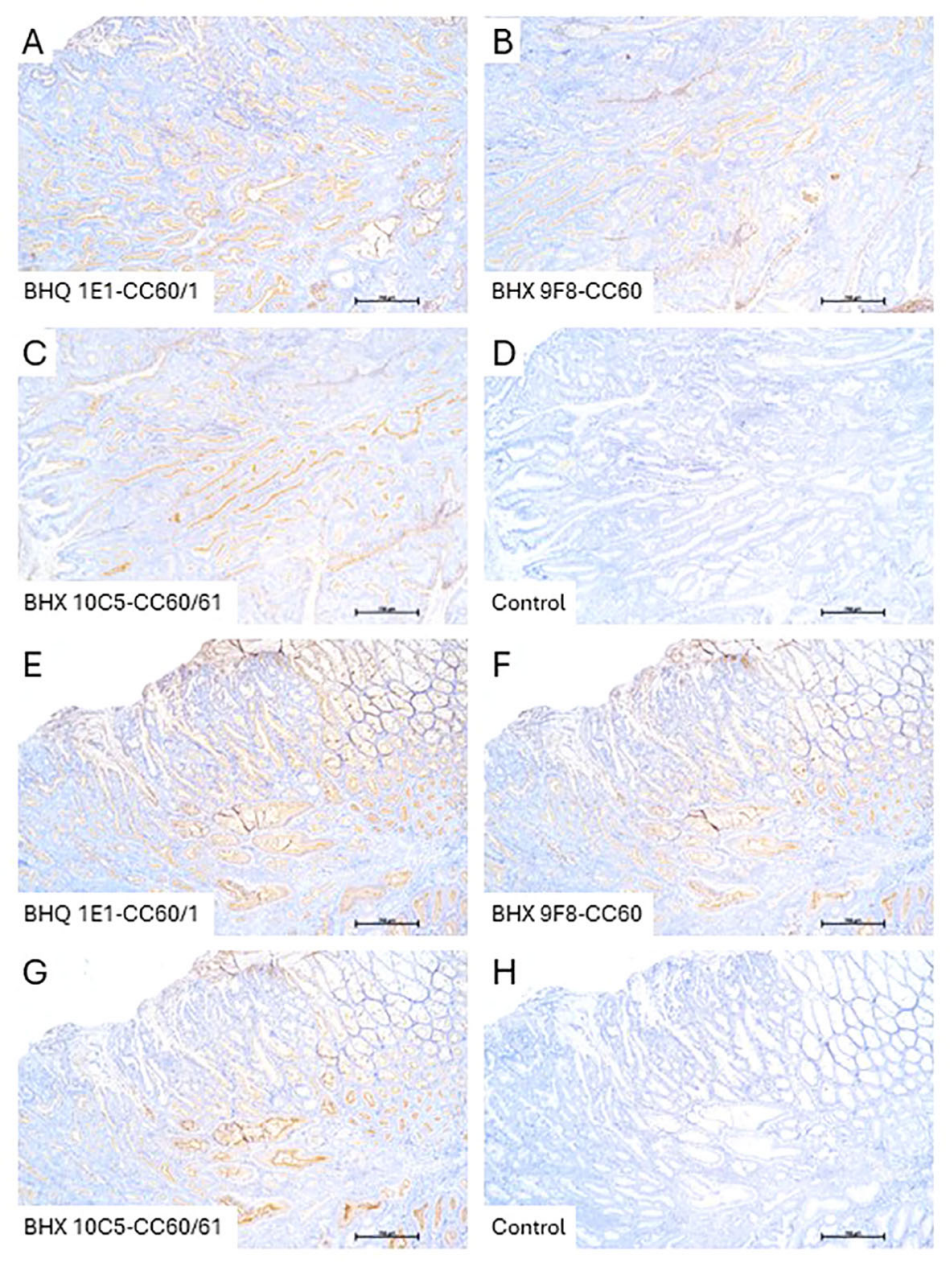
CEACAM60 is expressed in polyps of gene modified pigs. Paraffin-embedded polyp tissues obtained from 5 pigs with colonic polyps were examined for CEACAM60 expression by immunohistochemistry. Two polyps from two different pigs are shown. We obtained some considerable variation in CEACAM60 expression between different polyps and within individual polyp tissues using the monospecific anti-CEACAM60 mAb (BHX 9F8-CC60) and dual-specific mAbs anti-CEACAM1/CEACAM60 (BHQ 1E1-CC60/1) and anti-CEACAM60/CEACAM61 (BHX 10C5-CC60/61). The adjacent normal tissue showed uniform staining. Sample 1 **(A–D)** shows strong CEACAM60-specific antibody staining in almost all epithelial cells of the polyp, whereas sample 2 **(E–H)** shows pronounced heterogeneity of CEACAM60 expression among epithelial cells.

Since the porcine tumor model is still under development, it was not yet possible to assess the relevance of our data for the *in vivo* situation. So far, colon polyps have been generated in pigs, but no invasively growing carcinomas have been obtained. Examination of the colonic polyps revealed that CEACAM60 can be expressed by the transformed cells; however, expression levels varied considerably - both between different polyps and within individual polyps (**Figures 9A–H**). The staining patterns of all three mAbs (the CEACAM60-specific BHX 9F8 and the dual-specific mAb BHQ 1E1 (CEACAM60/CEACAM1) and mAb BHX 10C5 (CEACAM60/CEACAM61) appeared very similar in the polyps, indicating that the observed staining pattern is mainly due to the CEACAM60 expression or that CEACAM1 shares the same expression pattern in colonic polyps.

### 2.10 Detection of secreted CEACAM60

**Figure 10:**
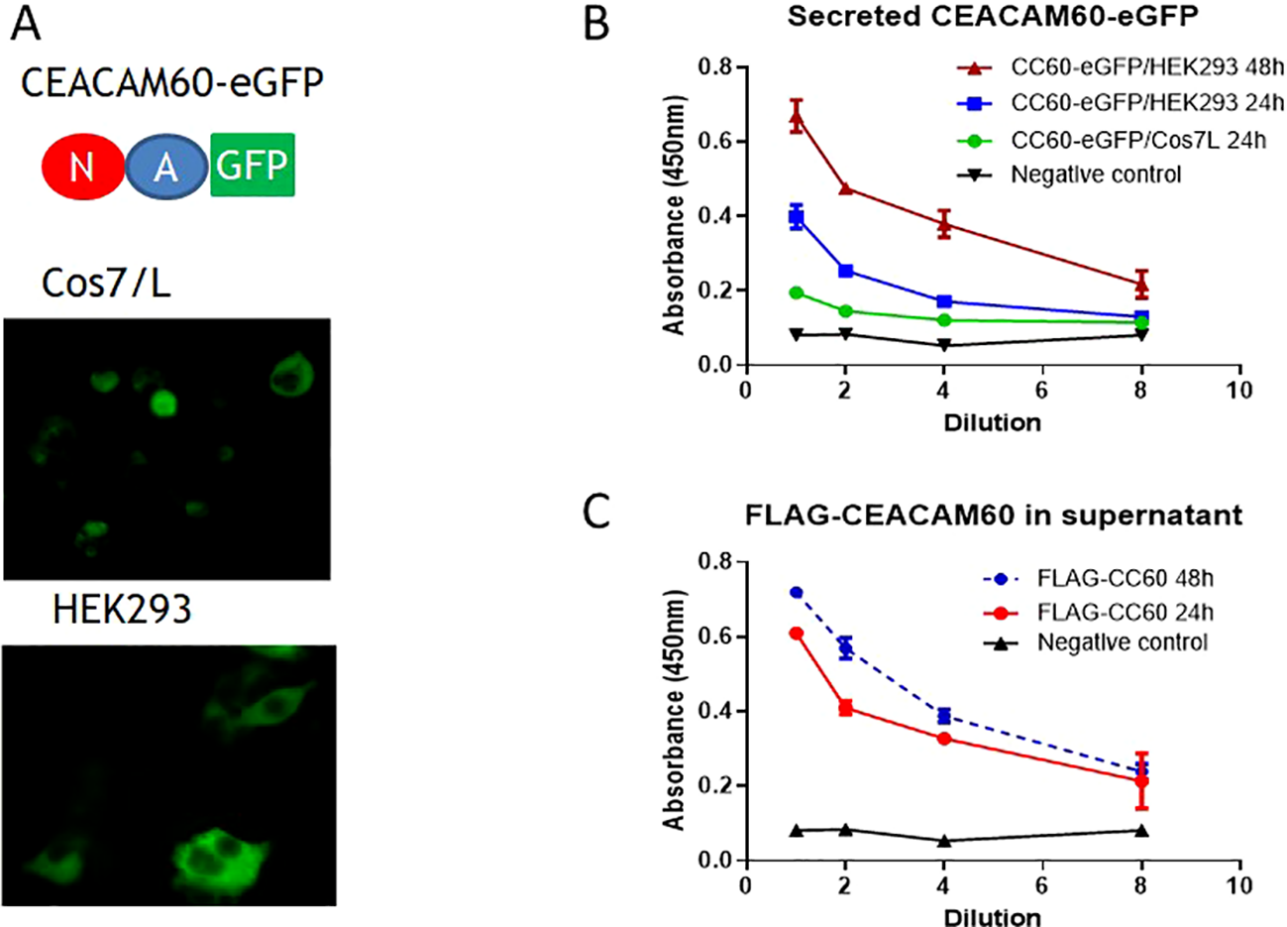
Sandwich ELISA for the detection of CEACAM60. To develop a sandwich ELISA for CEACAM60, a soluble eGFP−tagged CEACAM60 variant was generated. **(A)** Schematic representation of the construct and fluorescence microscopy of Cos7/L and HEK293 cells confirming its expression. **(B)** Dilution-dependent detection of soluble CEACAM60 in cell culture supernatants collected 24 and 48 h after transfection with the expression constructs. **(C)** Detection of spontaneously released FLAG−tagged membrane-bound CEACAM60 in the supernatant of transfected cells.

Reliable detection of soluble molecules is an important prerequisite for evaluating their potential as biomarkers. To enable detection of soluble CEACAM60, we established a sandwich ELISA based on two CEACAM60-specific mAbs. The capture mAb was derived from mouse (BHX 9F8) and the detection mAb (BHR 4F7) was derived from rat, allowing detection of the bound detection antibody using a biotinylated anti-rat secondary antibody. As a probe we generated a secreted CEACAM60 construct by replacing the TM encoding exon with the eGFP sequence, creating a soluble CEACAM60-eGFP fusion protein. This construct was transfected into Cos7 and HEK293 cells (**Figure 10A**). Supernatants of transfected cells were harvested at the indicated time points, and secreted CEACAM60 was detected by sandwich ELISA. Signal intensity increased depending on cell type and culture time (**Figure 10B**). We further analyzed how the GPI-anchored CEACAM60 is released from the cell surface under normal culture conditions. As shown in **Figure 10C** CEACAM60 was released into the culture supernatant, resulting in detectable levels of soluble CEACAM60 in the culture supernatants.

## 3 Discussion

The availability of whole-genome data from three different pig breeds enabled comprehensive analysis of the porcine *CEA* gene family. Our data showed that the conserved *CEACAM* genes are present in the pig genome and that the number of CEACAM1-related CEA family members is relatively small but comparable to the *CEA* gene family previously described in cattle (14). We identified four paralogs of CEACAM1 but no pregnancy-specific glycoproteins. Structurally, the porcine CEACAM1-related CEACAMs exhibit remarkable diversity, including an inhibitory CEACAM1 containing an ITIM and an ITSM, a GPI-anchored CEACAM60, a CEACAM61 harbouring an ITAM-like signalling motif, a putatively secreted CEACAM1, and two CEACAMs (CEACAM62, CEACAM63) with TM and very short cytoplasmic tails. From a phylogenetic point of view, we found coevolution of inhibitory and activating CEACAM receptors in the porcine CEA family as previously described for other species (26, 28, 29). Analysis of paired receptor expression (CEACAM1 and CEACAM61) revealed that CEACAM61 is preferentially expressed by neutrophilic granulocytes and monocytes, slightly differing from the granulocyte-specific expression pattern of human ITAM-bearing CEACAM3 (30, 31). Gene conversion is a predominant mechanism maintaining similarity among the ligand-binding domains of these paired receptors. We identified gene conversion as a frequent event in the porcine CEA gene family. Most remarkably, we discovered the first GPI-anchored CEACAM in a non-primate mammal. A recent blast search using the CEACAM60 membrane domain sequence detected related sequences (containing the stop codon) in all Suidae genomes but not in Tayassuidae species (pecaris) in databases, indicating that the GPI anchorage evolved after the divergence of Old World and New World pigs between ~15–30 million years ago (32). In humans four GPI-anchored CEACAMs exist, including carcinoembryonic antigen (CEA). Historically, CEA was the second serum tumor marker discovered in the mid-1960s for colorectal cancer, shortly after alpha fetoprotein (AFP) for liver cancer (12, 13, 33). In the following years it turned out that CEA is a member of a large gene family in humans, nowadays called the *CEA* gene family (34). CEA remains one of the most important tumor markers, particularly for colorectal cancer surveillance. More recently, CEA has gained attention due to its expression in nearly 100% of tumor cells in certain types of cancers, making it an ideal target molecule for tumor therapies. In recent years, numerous publications have explored CEA as a target antigen in experimental tumor therapies, particularly immunotherapies (35–37). GPI anchoring is pivotal for CEA’s utility as a serum tumor marker, as it enables continuous release from the tumor cell surface. After tumor invasion and loss of differentiation, the released CEA inevitably enters the patient’s blood plasma and can be measured in the serum. The exceptional stability of CEA as a relatively large glycoprotein of the immunoglobulin superfamily significantly increases its value as a tumor marker. In addition, the GPI anchorage linking CEA to the tumor cell surface is a prerequisite for its use as a target antigen in tumor therapies. Since CEA is primate-specific there is no non-primate animal model, which naturally express CEA and can be used to study the efficacy of tumor therapies that use CEA as a target molecule. In recent studies CEA transgenic mice, that were prone to get tumors in various tissues, were used to determine the impact of CEA as a target antigen (38, 39). Although CEACAM60 is not the porcine ortholog of CEA, it shares several notable features with CEA and may therefore function as a surrogate in pigs, providing important insights into CEA−based tumor therapies. In particular, the identical expression profile of CEACAM60 and CEA in the digestive tract may provide some information about the accessibility of colorectal tumor cells in CEA-targeted tumor therapies. Detection of CEACAM60 in the serum of tumor-bearing pigs would provide a simple tool for monitoring experimental CEACAM60-targeted therapies. This approach could substantially reduce the number of animals required in preclinical studies. To achieve this goal, we have established a sandwich ELISA to be able to measure soluble CEACAM60 in liquids. However, we are aware that this is only a first step to exploit the potential of CEACAM60 as a surrogate for CEA in serum. The establishment of pig models that develop invasive colon tumors, must prove, that tumors lead to constant levels of CEACAM60 in the serum of these animals, and verification that CEACAM60 can be reliably and specifically detected in pig serum by the established ELISA must follow. At present we could only analyse expression of CEACAM60 in colon polyps derived from APC1311/+ pigs, however, the impact of this investigation is limited, since the expression of CEA in human colon polyps is also very variable and they cannot be detected by an significant increase of the CEA serum level in humans (40, 41). Nevertheless, pigs are new promising animal models with several advantages over murine models, especially for colon cancer. For example, mutation in the human adenomatous polyposis coli (*APC*) gene causes lesions in the colon that often progress to metastatic cancer. In contrast, mice with a mutated *Apc* gene develop non-metastatic neoplasia in the small intestine (42). Since the physiology of the digestive tract of the omnivore swine is much more closely related to humans than that of rodents, pig models may have the potential to revolutionize preclinical colon cancer research. However, until now such a model is only on the way to be established (43). On the other hand, several tumor-bearing pig models have already been established, and it would be worthwhile to investigate CEACAM60 expression in these cancer models (44).

## 4 Material and methods

### 4.1 Datasets and nomenclature of genes

Sequence similarity searches were performed using the NCBI BLAST tools blastn http://blast.ncbi.nlm.nih.gov/Blast.cgi and Ensembl BLAST/BLAT search programs http://www.ensembl.org/Multi/Tools/Blast?db=core using default parameters. For identification of porcine CEACAM exons, exon and cDNA sequences from known CEACAM and PSG genes were used to search whole-genome shotgun contigs (wgs) databases limited to organism *Sus scrofa*. Hits were considered to be significant if the E-value was < e-10 and the query cover was > 50%. Once a whole genome shotgun (wgs) contig was identified that contained CEACAM-related sequences we confirmed manually the presence of the complete exon by the number of nucleotides and identification of CEACAM-typical splice donor or acceptor site sequences. Only sequences which were considered to be complete exons were used for further analyses. In a second step we used the identified exon sequences to search the database again in order to identify all existing paralogous CEACAM genes. Once we had identified individual exons, we predicted the gene structure based on the organization of known CEACAM genes. The location of different exons on the same contig was a prerequisite for considering that these exons belong to the same gene. Gene predictions were further supported by the identification of EST sequences and Transcriptome Shotgun Assembly (TSA) and/or predictions in genome builds at NCBI and Ensemble, if available. Short exons, like exons encoding cytoplasmic tails, were identified by alignments of downstream sequences of identified TM exons with cytoplasmic exon sequences of human and/or cattle CEACAMs. Sequence alignments for exon identification were performed using CLUSTALW (http://www.genome.jp/tools/clustalw/). For the identification of CEACAMs from other artiodactyls the following WSG data sets were used: Sus scrofa LUXX01 (95.5x; ST: Illumina HiSeq). The CEA gene family in swine is not well annotated; therefore, we adopted the nomenclature according to the one previously used for the CEA gene family of other mammals i.e. porcine CEACAM1 paralogs were numbered CEACAM60-CEACAM63. Gene names and corresponding sequences are summarized in **Supplementary Data 1**. The following databases were used for gene loci analyses: Sscrofa11.1 assembly and WSG sequence AEMK02000041.1.

### 4.2 Phylogenetic analysis and bioinformatics

Phylogenetic analyses based on nucleotide sequences were conducted using MEGA7 (45) or MEGAX (46). Sequence alignments were performed using Muscle implemented in MEGA. Phylogenetic trees were constructed using the maximum likelihood (ML) method with bootstrap testing (500 replicates) and the Tamura-Nei substitution model. Gene conversion was analyzed using the GENECONV program (version 1.81a) (47).

### 4.3 Cells and tissues

Different porcine tissue samples were collected from freshly slaughtered healthy pigs (Sus scrofa Cross-bred, n=3) and either flash-frozen in liquid nitrogen or stored in RNAlater (Invitrogen) until further use. Normal mucosa and polyp samples were collected from APC1311/+ pigs (n = 30) and stored at -80 °C for further molecular analyses. For histological and immunohistochemical analyses tissue sections were rinsed in cold phosphate-buffered saline (PBS) and fixed in 4% paraformaldehyde (Sigma-Aldrich, USA) for 24 hours at room temperature. After fixation, tissue specimens were transferred into 70% ethanol and then embedded in paraffin.

### 4.4 Reverse transcription-polymerase chain reaction

Total RNA extraction was performed using the RNeasy kit (Qiagen, Hilden, Germany). One microgram total RNA was used for cDNA syntheses by reverse transcription (RT) using the AMV Reverse Transcriptase (Promega, Mannheim, Germany). The RT product was amplified by polymerase chain reaction (PCR) with Taq polymerase (Fermentas). Enzyme reactions were performed as recommented by the manufacturers. After an initial denaturation step at 95 °C for 45 s, 35 PCR cycles (denaturation: 95 °C, 30 s; annealing: 60 °C, 1 min; extension: 72 °C, 1.5 min) and a final extension step at 72 °C for 15 min were performed. Primers used are summarized in **Table 1**. Eight microliters of each PCR were analyzed by electrophoresis on a 1.8% agarose gel and visualized by ethidium bromide staining.

### 4.5 Generation of anti-CEACAM60 mAbs

Anti-CEACAM60 mAb were generated by Genovac GmbH, (Freiburg, Germany) by genetic immunization. CEACAM60 cDNA was cloned from colon tissue of a healthy pig and sequenced by Sanger sequencing. The resulting sequence was provided to Genovac GmbH for immunization of mice and rats, according to their standard protocols. After cloning and selection of hybridoma cells the specificity of mAb binding was analyzed by flow cytometry.

### 4.6 cDNA cloning

Primers used for amplification of full-length cDNAs are shown in **Table 1**. For cDNA cloning of CEACAM61 the RT product was amplified by PCR with Easy-A High-Fidelity PCR Cloning Enzyme (Agilent) and analyzed by agarose gel electrophoresis. Specific bands were extracted from the agarose gel using QIAEX II Gel Extraction Kit (Qiagen). The PCR products were cloned using the StrataClone PCR Cloning Kit (Agilent).

CEACAM60 cDNA without the leader sequence was amplified using primers 639 and 640 (**Table 1**) yielding a 932 bp product, which was cloned into the pFLAG-CMV™-3 Expression Vector using HindIII and XbaI restriction sites.

Plasmid DNA isolated from various clones were analyzed by PCR and sequencing. Nucleotide sequencing was performed with the BigDye Terminator Cycle Sequencing Kit (PE Applied Biosystems, Weiterstadt, Germany).

### 4.7 GPI linkage prediction and phospholipase cleavage of GPI anchor

For prediction of GPI anchorage three different prediction programs were used: PredGPI (http://gpcr2.biocomp.unibo.it/gpipe/pred.htm), big-PI-Predictor (http://mendel.imp.ac.at/gpi/cgi-bin/gpi_pred.cgi) and GPI-SOM (http://gpi.unibe.ch/). For determination of phospholipase C sensitivity of CEACAM60 anchorage CEACAM60 cDNAs were cloned into the pFLAG CMV3 vector. pFLAG CMV3-CEACAM60 and pRc/CMV-CEA plasmids were used to transfect Cos7/L-cells using the Lonza Amaxa Cell Line Nucleofector Kit V (Lonza Amaxa). 48 h after transfection cells were harvested, washed and suspended in 0.5 ml phosphate-buffered saline (PBS). Cell suspensions were incubated with 0.1 U/ml phosphatidylinositol-specific phospholipase C (PI-PLC) isolated from *Bacillus cereus* for 50 min at 37 °C. Thereafter cells were washed and analyzed by flow cytometry.

### 4.8 Flow cytometry

1x10^6^ Cos7 cells were transfected using the Nucleofector Kit V (Lonza Amaxa). 1x10^5^ transiently transfected Cos7 cells were stained with murine anti-FLAG mAb (clone M2, Sigma-Aldrich) as a primary mAb and a goat anti-mouse immunoglobulin G (IgG)-phycoerythrin (PE) as secondary antibody. Flow cytometry was performed with the MACSQuant Analyzer and the “MACS Quantify” software. Each mAb was tested at least in two independent experiments with similar results. Flow cytometry of porcine peripheral blood cells was performed with whole blood from Sus scrofa breed, Landrace (n = 5). 50 µl of full blood was used and erythrocytes were lysed with RBC lysis buffer (Biolegend). White blood cells were stained with the following antibodies. Murine anti-FLAG mAb (SIGMA, Taufkirchen); PE-coupled anti-mouse IgG1 mAb, clone A85-1, (BD Biosciences, Franklin Lakes, USA); anti-rat PE (donkey polyclonal, Jackson Immunoresearch); 6D10 APC (BioRad #MCA2599A647); porcine-specific anti-CD14 FITC mAb MIL2 (BioRad #MCA1218F); anti-CEACAM60 (BHX 9F8); anti-CEACAM1/CEACAM60 (BHQ 1E1); anti-CEACAM61/CEACAM60 (BHX 10C5) (all characterized in this study). Labelled cells were analyzed at the BD FACSymphony™ instrument and visualized with the FlowJo software.

### 4.9 Immunohistochemistry

Immunohistochemistry was performed from tissues of four animals from two different breeds (crossbreed and minipigs).

Five‐micrometer cryostat sections of colon and jejunum tissues were air‐dried and fixed in acetone for 10 min. Endogenous peroxidase activity was blocked with 10% methanol and 3% H_2_O_2_. Immunohistochemistry was performed using the avidin-biotin complex (ABC) method. The following monoclonal rat antibody was used: rat mAb, BHR 4F7 (anti‐CEACAM60). As secondary reagents we used anti-rat IgG biotin-conjugated goat antibody followed by streptavidin horseradish peroxidase conjugate (DakoCytomation). For visualization, diamino-benzidine (DAB) substrate solution was applied, followed by counterstaining with hematoxylin.

Serial 4-µm sections were prepared from paraffin-embedded tissues and mounted on 3-aminopropyltriethoxysilane-coated slides. After deparaffinization in xylene and rehydration through graded alcohol series, heat-mediated antigen retrieval was performed in 0.01 M sodium citrate buffer (30 min, 97 °C). Endogenous peroxidase activity was blocked with 3% H_2_O_2_ for 5 min, and nonspecific binding was reduced by incubation in 1% bovine serum albumin (BSA)/PBS for 1 h at room temperature). Sections were incubated overnight at 4 °C with mouse anti-CEACAM60 (BHX 9F8), dual-specific anti-CEACAM60/CEACAM61 (BHX 10C5), or rat anti-CEACAM1 (BHQ 1E1) mAbs (10 µg/ml in 1% BSA/PBS). Isotype-matched Ig mAbs served as a negative control. Biotinylated rabbit anti-mouse or anti-rat secondary antibodies (Dako, Germany; 1:200 in 1% BSA/PBS) were applied for 1 h at room temperature, followed by VECTASTAIN ABC reagent (Vector Laboratories, USA; 30 min). Staining was visualized with DAB and stopped in distilled H_2_O. Sections were counterstained with hematoxylin, dehydrated, and mounted with Xylene Substitute Mountant (Thermo Fisher Scientific, Germany).

### 4.10 Sandwich enzyme-linked immunosorbent assay

To detect secreted CEACAM60 in culture supernatants, a sandwich ELISA was established. A CEACAM60 construct lacking the TM was generated by cloning the truncated CEACAM60 cDNA sequence into the pEGFP−N3 vector, resulting in an eGFP−tagged, CEACAM60 fusion protein used during assay development. Two micrograms of plasmid DNA were transfected into 1×10^6^ Cos7L or HEK293 cells cultured in six−well plates containing 2 mL of medium per well. Supernatants were collected after 24 h and 48 h of incubation, centrifuged at 2500 rpm for 10 min to remove debris, transferred to 1.5 mL cryovials, and stored at 4 °C. For the sandwich ELISA, 5 µL of purified mouse mAb BHX 9F8 (2.4 mg/mL) were diluted in 5 mL of coating buffer (carbonate/bicarbonate buffer, pH 9,5) and used as the capture antibody solution. Each well of a Nunc 96−well plate was coated with 50 µL of this solution and incubated overnight at 4 °C. Plates were then blocked with 200 µL PBS/BSA blocking buffer for 2 h at room temperature and washed twice with ELISA washing buffer (PBS: 0.1% BSA: 0.05% Tween-20). Subsequently, 50 µL of supernatant (diluted 1:1) were added per well. Control wells containing culture medium only were included to determine background signal, and serial dilutions of the supernatants were prepared before addition to the wells. The plate was incubated for 3 h at room temperature, and washed four times with ELISA washing buffer. For detection, 10 µL of rat mAb BHR 4F7 hybridoma supernatant were diluted in 5 mL of PBS/3% BSA, and 50 µL of this solution were added to each well, incubated for 1 h at room temperature, and washed six times. Next, 1 µL of anti−rat biotin−conjugated antibody was diluted in 5 mL of PBS/3% BSA, and 50 µL were added per well, followed by a 1 h incubation and six washes. Following the ABC protocol, 50 µL of streptavidin−horse radish peroxidase (1:1000) were added for 30 min at room temperature and washed eight times. Finally, 100 µL of slow TMB substrate solution (ThermoFisher Scientific) were added for color development (30 min, room temperature), and the reaction was stopped with 100 µL of 0.5 M sulfuric acid. Absorbance was measured at 450 nm using an Infinite F200 Pro multimode microplate reader.

## Data Availability

The datasets presented in this study can be found online repositories at NCBI. The newly sequenced mRNAs have the following GenBank accession numbers: BankIt3072829 CEACAM1 PZ261328; BankIt3072829 CEACAM60 PZ261329; BankIt3072829 CEACAM61 PZ261330.
