# Supplementary material for: The significance of CEACAM60, a carcinoembryonic antigen (CEA) homolog, as a tumor antigen in the porcine cancer model
Source: Front Immunol. 2026 May 13;17:1813834. doi: 10.3389/fimmu.2026.1813834 (PMC13212111; doi:10.3389/fimmu.2026.1813834)
Supplement: Supplementary file 1 [file DataSheet1.docx]

Supplementary Material

# Supplementary Data

**Supplementary File 1 – predicted and confirmed coding sequences of porcine CEACAMs**

(Exon sequences are indicated by different colors)

**> CEACAM16 [Sus scrofa] (predicted)** **ENSSSCT00000034675.4**

ATGGTGCTGACCGGGTACAGCTGGCTTCTCCTCAGTGCTGCCTTCCTGAGCACGGGGGCCGAGATCACCATCACCCCCGAGCCCACCCGGCCAGCCGAGGGGGACAACATCACCCTGGCCGTCCAGGGGCTTTCGGGGGAGCTGCTTGCCTACAACTGGTACGCGGGGCCCACGCTCAGCCTGTCTTACCTGGTGGCCAGCTACATCGTAAGCACAGGCGACGAGACCCCCGGCCCGGCCCATACGGGGCGGGAGGCTGTGCGCCCTGACGGCGGCCTGGACATCCAGGGCGTCCTGCCCCGGCACTCGGGCACCTACATCCTGCAGACTCTCAACAGGCAATTTCAGACGGAGGTGGGCTACGGACACTTGCAGGTCTATGAGATCCTGGCCCAGCCTGTGATCATGGCCAATAGCACAGCACTGGTGGAGCACCGGGACACCCTGCACCTGACATGCAGCAGCCCCAGCCCTGCTGAGGTCCGCTGGTTCTTCAACGGTGACGCCCTGCCCATCTCCGTCCGCCTTGGCCTGTCCCCCGACGGCCGGGTCCTGATCCGGCACGGTGTCCGGAGGGAGGAGGCCGGAGCCTACCAGTGTGAGGTCTGGAACCCGGTCAGTGTCAGCCGCAGCAAGCCCATCAACCTGACCGTGTACTTTGGCCCAGAGCGCGTGGCCATCCTCCAGGATTCCACCACCCGCACGGGCTGCACCATCAAAGTCGACTTCAACACGTCACTCACGCTGTGGTGCGTGGCCCGGTCTTGCCCGGAGCCCGAGTACGTGTGGGCCTTCAACGGGCGGGCCTTAAGGAACGGCCAGGACCACCTCAACATCAGCAGCATGACAGCGGCCCAGGAGGGCACGTACACGTGTATCGCTAAGAACCCCAAGACTCTGCTTTCTGGATCCGCCTCAGTGGTGGTCAAACTTTCCGCGGCAGCTGTCGCCGTAACAATTGTGCCCGTGCCAACCAGGCCGATGGAGGGCCAGGACGTGACGCTGACCGTACAGGGCTACCCCAAGGACCTGCTGGTCTACGCCTGGTACCGGGGGCCTGCTGCGGAGCCCAACCGACTGCTTAGCCAGCTGCCTTCGGGGAACTGGATCGCAGGCCCTGCGCACACAGGCCGGGAGGTGGGCTTCCCCAACTGCTCGCTGCTGGTACAGAAGCTGAACCTCACGGACGCAGGCCGCTACACGCTCAAGACCGTCACGCTGCAGGGCAAGACTGAGACGCTGGAAGTGGAGCTGCAGGTGGCCCCCCTGGAGTAG

**> CEACAM18 transcript 1 [Sus scrofa] (predicted)**

ATGGACCTTTCCAGACAGAGGTGCAGACTCTGGAAGGAACTAGTCCTTGTGGCCAGCCTGCTAGCCTTGGGGATCTGCCAGGCCTCTGGCCAAATCGACATCAACCCAGACTCACTGATAGGAGTGGAAACATACCGGACCCGCCTGGTCCTTGAAAACGGCCCTGAAGATGCCCAGGCCTATAGCTGGCACCGCGGCGCAAATGACACTGAGGAAAACATGATTGTCAGCTACAACGTCACCTCTGATTCCCAGCAGAACGGGCCCATGTTCACCGGCCGGGAAAGTGTGCTGAAGACGGGTGACCTGCACATCAGGGGGTCTCAGTTAAATGACACAGGGAACTACACAGTGCGGGTGGACGCCATCAATGGGACCCAGAGAGCAACTGGCTGGCTCAAGATTCAAGAATTGGAAATCCCTGGGATCTCAGTCAACGCCACTTCTGTGGTCGAGGACATAGATTCCGTGGCTGCCATCTGCTACACCAATGACACCAACGTCATGTGGTATGTGAATTCTGCACCAGTGTCCAGCAATAACCGGATGACAATCTCCCCAGACACCAAGACACTCATTATCCAAAGGATTGAACGCTTTGATTCACCACTTCAGTGTAGCATAGAAATCATCCCGGAGATTTTTGGGAGAAGTGAGCTGATCCAGCTGACGGTAGCCTATGGGCCCTACGGCATGACCCTCACCAGCATGCCCAACCACCTCAGCGGCGTCGTGTCTGCCGAGATCGGCACCCCGGTGGAGATGAAGTGCACAGCCTATTCCAGACCGGAATCCCAGTACCGCTGGTTCCACAACGGCTCCCTGCTGAGCTTCTCAGGGGAAAACATTAGCCTCCCAAGCCTGACCTGGGACCAGATGGGCAGCTACAGATGCGTCGTGGGGAACTCCGTCACCCAGCTGACCTTCTACCAGAGACTCCAGGTCCAGGTGCCCTGGCGCCCCAAGCCTGCTCAGAAAAGAGTCTTCACCGTCTCAGGATCCTATGTGGTGGTGCTTATCGTAGTGACAGTCCTGGGCGGTGTCGTCTTTTGTGCAACCCTGATCTACATTCTGTTCAAACACTACTCCACCAGGACAAATAGGCTTATATGA

**> CEACAM18 transcript 2 [Sus scrofa] (predicted)**

ATGGACCTTTCCAGACAGAGGTGCAGACTCTGGAAGGAACTAGTCCTTGTGGCCAGCCTGCTAGCCTTGGGGATCTGCCAGGCCTCTGGCCAAATCGACATCAACCCAGACTCACTGATAGGAGTGGAAACATACCGGACCCGCCTGGTCCTTGAAAACGGCCCTGAAGATGCCCAGGCCTATAGCTGGCACCGCGGCGCAAATGACACTGAGGAAAACATGATTGTCAGCTACAACGTCACCTCTGATTCCCAGCAGAACGGGCCCATGTTCACCGGCCGGGAAAGTGTGCTGAAGACGGGTGACCTGCACATCAGGGGGTCTCAGTTAAATGACACAGGGAACTACACAGTGCGGGTGGACGCCATCAATGGGACCCAGAGAGCAACTGGCTGGCTCAAGATTCAAGAATTGGAAATCCCTGGGATCTCAGTCAACGCCACTTCTGTGGTCGAGGACATAGATTCCGTGGCTGCCATCTGCTACACCAATGACACCAACGTCATGTGGTATGTGAATTCTGCACCAGTGTCCAGCAATAACCGGATGACAATCTCCCCAGACACCAAGACACTCATTATCCAAAGGATTGAACGCTTTGATTCACCACTTCAGTGTAGCATAGAAATCATCCCGGAGATTTTTGGGAGAAGTGAGCTGATCCAGCTGACGGTAGCCTATGGGCCCTACGGCATGACCCTCACCAGCATGCCCAACCACCTCAGCGGCGTCGTGTCTGCCGAGATCGGCACCCCGGTGGAGATGAAGTGCACAGCCTATTCCAGACCGGAATCCCAGTACCGCTGGTTCCACAACGGCTCCCTGCTGAGCTTCTCAGGGGAAAACATTAGCCTCCCAAGCCTGACCTGGGACCAGATGGGCAGCTACAGATGCGTCGTGGGGAACTCCGTCACCCAGCTGACCTTCTACCAGAGACTCCAGGTCCAGGTGCCCTGGCGCCCCAAGCCTGCTCAGAAAAGAGTCTTCACCGTCTCAGGATCCTATGTGGTGGTGCTTATCGTAGTGACAGTCCTGGGCGGTGTCGTCTTTTGTGCAACCCTGATCTACATTCTGTTCAAACACTACTCCACCAGGGGATAG

**> CEACAM19 [Sus scrofa] (predicted)**

ATGGAGATTCCTGCTGGGGCCCAGCAGCCCTTCCCAAGGGGCCTCCTGCTCTCAGCCTCAATCCTGGCCCTCTGGGTCCCCCAAGGCTCCTGGGCAGCCCTCCGCATCCAGAAGATTCCAGAGCAGCCTCAAACGAACCAGGACCTTCTCCTGTCTGTCCAGGGCATCCCAGGCTCCTTCCAGGACCTCACCTGGTACCTGGGGGCAGAGGCCGATGGCGGGACGATGCTCTTCACCTACATTCCGGAGCTACTGCGGCCCCAGAGGGACAGCAGCTCCATGAAGCAGCGCGACATCGATGGCTTCCCCAACGGCTCCATGCTGCTGCGTCGTGCCCAGCCCGCTGACAGCGGCACCTACCAGGTGGCTGTCACCATCAACCCTGCATGGACCATGCGGGCCAAGACTGAAGTCCAGGTGGCTGAAAAGCCGAAGGAGCTGTCCATTACAAACCTGCCCATGAGTGCTGGGATCGTGGCTGCCATCATCATTGGGTCCCTTGCCACCGGCTCTGTCTTCGTCGGCAGCATTGCCTACCTCCTGGTGACAAGAGGCTGGAGGGCCCAGAGCCACAGGATGATGGCCTCGGAGAAACCAGAGCTGGGCCCCCATCATCATGCCGGCGACAACAACATCTATGAAGTGATGCCGTCTCCGGTCCTCCTGGTGTCCCCTCTCAGTGACACGGGGTCCATGAACGCCATGGCGCCGCCGCCCCTGCCCCCGCCCCCGCCCCCTGCGCCGGAGCCGGAGAACCAGCCCTACCAGGACCTGCTGAATCCGGACCCTGCCCCTTACTGCCAGCTGGTGCCAGCCCCCTGA

**> CEACAM20 [Sus scrofa] (predicted)**

ATGGCGTCCACTGCCCTCTGGGCCCACCACTGGGCAGGACTCCTGCTCTCAGCCTCGCTTTTGACCACATGGAGCCTGCCAGCTGCAGCCCAGCTCACCCTTGGTGACAGACTGCCTAAAGCCACCCGAAGCTCACTGGCCAAGCCCACCATTGCAGTCAGCCAGGGCACTGCCATAGAGCACAGGGAAGGGGTGAGCTTCTACTGTGACACCAAGGACGTAAATATTACCATCTACTGGGTCTCCAACAATCACCCCTTGAAGTTCGATGAGCGCATGTGGCTGTCCACGGATCGCAAGAACCTCACCATCCTCACTGTCCAGCGGGAGGATGCAGGGACTTACCAGTGTGAAGTTTGGGGTGTCCTCCAGGTCCAGAGCAGCAACCCCACCTTCCTGATCGTGTACTATGGTCCTGACCCTGTTGAAATCAAGTTGGAGCCTGGTGTACCCAGTGGGGAGGCAGTTGAGGTGATAGAGGGCTCCAACTTGACCTTCTCGGTGGAAACACTGTCTCATCCACACCCTGACTATTCATGGTTTTTCTCTAATGACTCCAAGCCCATCACGAGCTTGAGTTCCACCACGAGCACGTTTACCCTCCATGCTGCATCCAAGGAACACGAGGGCCTGTACAGGTGCTTGGTGTCCAACAAGGCCACCAACCTGTCCCGTCTGGGTGCCCTTAAAGTCCGCGTCCTTGAAAGAGTGACCAAGCCTTGCATCACATCCCCAAACCTGAATCTTGTGGAGAATGCCAGCCTGGTGGTCCTGACCTGCCAGACCAGCCATGAGGGGGTTGGAGTCCAGTGGTTCTTGAGGGGCCAGCCCCTCCTGCCCAGCCCGCACCTGGTGCTGTCAGCTGACAACAGGACCCTGGTCATCCACGGCCTCCGGCGAGATGACGTGGGGCCCTACGAGTGTGAGGTCTGGAACTGGGGCAGCAGGGCCCGGAGCGACTCCTTCAGGCTGAACATCAGCTATGGCCCTGATCGAGCGGACATCACCAGGGGCCCGGCATCCGAGGCGGTCAGCACCATCAAGGCAGAGTTCAACTCCAGCTTGACCCTGCAGTGTCGGGCTGAATCCCAGCCAGATGCTGAGTTTCACTGGACCCTTGAACACTCCACCAGTGTGTGGACGGGGGAGCAGCTGATCATCGAGGCCCTGACCTGGGAACACCAGGGGACCTACAACTGCGTGGCTTTTAACTCTCTGACCCACCTGGCCAGCTCCGCCTCCGTCCGGGTCAGGGTGATGGGTCCCCAGTTGTCCCAGTCTATAGGGGCCATCACTGGCATTACCATCGGGAGTGTGGCTGTTGTTGCCCTGGCCACAGGGCTGGCCTGTTTCCTCTACATCAGATATGCCAAAGGGTCCTCAAGGAGAACAACAGAGGACCTCATCCTTGAGGCCAGGACACCCACCTCTGAAACGACTCATCCTGCAGAGCCTGGTCGCAACTGGCCCATGCCCACGTATGCCAATGTACCCAACACTCAAGAACAGGTCCGAGTCAAAAAGGTGCTGACACAGGACCCTCCAGGGGAGTTCCATGAGAAGGAATCATCTTCAGCGGTCCATGGGGGCTATTCTCGTGGCCCCAGGAAGCCACAGCCCAAACTGGCATCGGATCCATCGGTCCCAACTCTACCAAAGGGAAACACAGAGTCAAACTATGAGGTGCTGGTGAATCCAGAGCACAACCTTTACTGCCACATCAACTCCTCCGTCTAA

**> CEACAM1 [Sus scrofa] (confirmed)**

ATGGAGCCCCCCTCAGCCCCTGCCCACAGAGGGCATGTCCCCTGGCACAGGCTCCTGCTGGCAGTCTcactcctaagCTTCTGGAACTTGCCCGCCACTGCCCAGATCACTATTGAATCAGTGCCCTTCAATGTCGCAGAAGGAAGCGATGTTCTTCTACTTGCCCACAATGCGACAGAGGATACTCTAGGCTACAGCTGGTACAGAGGAGAAAGAGTAGAGAACAACCAACTAATTGTAACATACAGAATAGACACTCAAGCATATACCCAAGGACCTACATTCAGTGGTCGAGAGAGAATCTACCCCAATGGATCCCTGCTGTTCCAGAATGCCACCCAGAATGACACAGGATACTACACTCTGATGGTTACAAAGAATGATTTACAGAGAGAAATTGTAACTGGACTACTCCGTGTATACCCGGTGTTACCCAGGCCCATCATCACCAGCAACAACCCCAACCCCGTGGAGCACGAGGACACTGTGGTGTTAACATGTGAACCTGAGACTCAGCACACAACCTACAGGTGGTGGATCAACAACCAGAGCCTCTCCAACAGCACCAGGCTGCAACTGTCTGAGGACAACAGGACACTCACTTTATTCAACGTCACCAGAAATGACACAGGACCCTATGAGTGTGAAACCCAGAACCCAGTGAGTGCCACCCGCAGTGACCCATTCACCCTGAATGTTATCCACCCAGTGGCAAAGCCCATCGTCGAAGCCAACAGCACCACCGTCACAGAACACGAGGACACTGTGGTCCTGAAATGCCTCACAAATGACACCGGGGTCTCCTTACGCTGGCTCTTCAATGGCCAGAGCCTACTGCTCGCAGAGAGGATAAAACTGTCCCAGGACAATGGCACCCTCACCATAGAGCCCGTCAGGAGGGAGGATGCTGGGAATTATCAGTGCGAGGCCTCCAACCTGGGCAATTCCAACAAAAGTGACCCCCTCAGGCTGGATGTGAAATTTGATTCAGCACCAGGAAGTTCTTCTGGCCTCTCAGGTGGTGCTATTGCTGGCATCGTGATCGGAGTCCTGGCTGGGGTAGCTCTGATAGCAGGCCTGGTGTATTTCCTGTTTATCAGAAAAATTGGAGGGGCAAGTGACCAGCGCCAACTCACAGAGAACAAATCCTCAGACTTCAACCACATTCAGGACCACTCTGACAAACTGTCCAAGAAGATTGATGAAGTTACATACTCTTCCCTGAACTTCAATGCCCAGGAATCAAAGAAACCTACTTCAGCACTTCCATCTCCATCAGCCACAGAAACAGTTTATTCAGAAGTGAAAAAGAAGTAA

**> CEACAM60 [Sus scrofa] (confirmed)**

ATGGAGCCCCCCTCAGCCCCTGCCCACGGAGGGCGCATCTCCTGGCACAGGCTTCTGTTGGCAGTCTCACTCCTAAGCTTCTGGAACCTGCCCGCCACTGCCCAGATCACTATTGAATCAGTGCCCTTCAAAACTGCAGAAGGAAGGGATGTTCTTCTACTTGCCCACAATGCGACAGAGGATACTCTAGGCTACAGCTGGTACAGAGGAGAAAGAGTAGAGAACAACCAACTAATTATATTATATAGAGTAGACATTCAAGCAAATACCACAGGGCCTGCATACAGTGGTCGAGAGATAATCTACCCCAATGGATCCCTGCTGTTCCAGAATGCCACCCAGAATGACACAGGATACTACACTCTGATGGTTACAAAGAATGATTTACAGAGAGAAATTGTAACTGGACTACTCCGTGTATACCCACCAGTGACAAAGCCCATCATTGAAGCCAACAGCACCACCGTCACAGAACAAAAGGACACCGTGGTCCTGACCTGCATCATAAATGACACTGAGGTCTCCATACGCTGGCTCTTCAATGGCCAGAGTCTACTGCTCACAGAAAGGATAAAGCTGTCCCAGGAAAACAGCACCCTCACCATAGAGCCCGTCAGGAGGGAGGATGCTGGGAATTATCAGTGCGAGGCCTCCAACCCAGGAAATTCCAGCCTAAGTGACCCTCTCAGGCTGGATGTGAAATTTGATTCAATACCAGGAAGTACTTCTGGCGTCTCAGATGGTGGTATTGCTGGCATTGTGATTGGAGTCCTGGCTGGGGTAGCTCTCATAGCAGGCCTGGTGTAA

**> CEACAM61 [Sus scrofa] (confirmed)**

ATGGAGCCCCCCTCAGCCCCTGGCCACAGAGGGCATGTCCCCTGGCACAGGCTCCTGCTGGCAGTCTCACTCCTGACCTTCTGGAACCTGCCCACCACTGCCAAGATCACTGTTGAATCAGTGACCTTCAATGCTGTAGAAGGAAGGAACATTCTTCTACTTGTCCACAATGCTATAGAGAATATTCTAGCCTACCACTGGTACAGAGGAGAAAGAATAAGGAACAGCCAACTAATTGCATCATATGAAGTAGACCGTCAAGTAACTATCACAGGACCTGCACACAGCGGTCGAGAGATAATCTACCCCAATGGATCCCTGCTGTTCCAGAAGGTCAACCAGAACGACACAGGATACTACACCCTGAGCATTATATACAATGATTTACAGACCGAAAGTGTAATCAAACAGCTCTACGTATACCCACCAGTGGCAAAGCCCATCATCGAAGCCAGCAACACCACCGGCACAGAACACGGGGTCACCGTGGTCCTGAAATGCCTCACAAATGACACCGGGGTCGCCATCTCCTGGTTCTTCAATGGCCAGAGTCTACTGCTCACAGAGAGGATGGAGCTGTCCCAGGACAACAGCACCCTCACCATAGAGCCCGTCAGGAGGGAGGATGCTGGGGATTATCAGTGCGAGATCTCCAACCTCATCGGTTCCAGTAAAAGTGATCCCTCCAGGGTGGATGTGATCTTGCAAGAAAATGGCCCAGTCCTCAGTGTGGGGGCCATTGTTGGCATTGTGATTGGGGTCCTGGTTGTGGTGGCTCTGGTGGCTGCCCTGGGATGTTTCATTTTCCTCGCAAGGATTCGCAGCCCTGGTTGCCTGCAGAACAGCAGAGAAAACTGGTACATAGAGTCAACACCGGGATATGGTCCCTCCAGCAGCTCTCTCTCCCCGGCCTGCCTATCTGACCGCATGCCAGAGGTTCCCATCTACCAGGAATTAGTACACCCCGACACAGACATTTACTGTGGGATGGACCACAAAGCAGATGTGGCTTCTTAG

**> CEACAM62 [Sus scrofa] (predicted_TSA evidence)**

ATGGAGCCCCCCTCAGCCCCTGCCCACGGAGGGCGCATCTCCTGGCACAGGCTTCTGTTGGCAGTCTCACTCCTGACCTTCTGGAACCTGCCCACCACTGCCCAGATCACTGTTGAATCAGTGACCTTCAATGCTGTAGAAGGAAGGAACATTCTTCTACTTGTCCACAATGCGACAGAGAATGTTCTAAGCTACCACTGGTATAGAGGAGATACAGCAGAGGAAAACCAACTAATTGCATCACATGCAGTAGACTTTCAACTAACTATCAGAGGACCTGCACACAGCGGTCGAGAGATAATCTACCCCAATGGATCCCTACTGTTCCAGAACGTCACCCTGAGAGACTCAGGAAACTACACTCTGATGGTTACAAAGAATGATAGACAGAAAGAAATTGTGACTGGACAACTCCGTGTATTCTACCCAGTGGCAAAGCCCATCGTCGAAGCCAACAGCACCACCGTCACAGAACACGAGGACACCGTGGTCCTGAAATGCCTCACAAATGACACCGGGGTCGCCATCTCCTGGTTCTTCAATGGCCAGAGTCTACTGCTCACAGAGAGGATGGAGCTGTCCCAGGACAACGGCACCCTCACCATAGAGCCCGTCAGGAGGGAGGATGCTGGGCATTATCAGTGCGAGGCCTCCCACCTGGGCAATTCCAGCAAAAGTGACCCCCTCAGGCTGGATGTGAAATATGATTCAGCAACTGGCCTCTCAAGTGGTGCTGTTGCCGGGATCGTGACTGGAGCCCTGGCTGGGGTAGCTCTGATAGGAGCCTTGGTGTATATTCTGTGTGTGATATAG

**> CEACAM63 [Sus scrofa] (predicted_TSA evidence)**

ATGGAGCCCCCCTCAGCCCCTGGCCACAGAGGGCATGTCCCCTGGCACAGGCTCCTGCTGGCAGGCTCACTCCTAACCTTCTGGAACCTGCCCACCACTGCCCAGATCACTATTGAATCAGTGCCCTTCAATGCTGTGGAAGGGACAAGTGTTCTTCTACTTGTCCACAATGTGACAGAGAATATCCTAGACTACTGCTGGTACAAAGGAGAAAGGGTAGAGACCAACCAGTTAATCGCATCATGTAGAGTGGACGCTCAAGCAAATACCCCAGGGCCTGCACAGAGCAGTCAAGAGACAATCTACCCCAATGGATCCCTGCTGTTCCAGAAGGTCGCCCAGAGTGACACAGGAAACTACTACACCCTACTTGCCACAAAGAGAGATAATCAGACTGAAAGTGTGACTGGACAACTCCGTGTGTACCCGGTGTTACCCAGGCCCGTCATCACCAGCAACAACCCCAACCTCGTGGAGCACAAGGACACTGTGGTGTTAACATGTGAACCTGAGACTCAGCACACAACCTACAGGTGGTGGATCAACAACCAGAGCCTCCCCAGCAGCACCAGGCTGGACCTGTCCAAGGACAACAGGACACTCGCTTTAGTCAGCATCAAAAGGAACGACGCAGGACCCTATGAGTGTGAAACCCAGAACCCAGGGAGTGCCGCCTGCAGTGATCCATTCACCCTGAATGTTATCCACCCAGTGGCAAAGCCCATCGTCGAAGCCAACAGCACCACCGTCACAGAACACGAGGACACCGTGGTCCTGAAATGCCTCACAAATGACACCGGGGTCGCCATCTCCTGGTTCTTCAATGGCCAGAGTCTACTGCTCACAGAGAGGATGGAGCTGTCCCAGGACAACGGCACCCTCACCATAGAGCCCGTCAGGAGGGAGGATGCTGGGCATTATCAGTGCGAGGCCTCCCACCTGGGCAATTCCAGCAAAAGTGACCCCCTCAGGCTGGATGTGAAATGTAAAAAAAAAACCTGCCCAACCTTCAGTGTGGAGGTCATTATCCTCATCGTGATTGGGGTCCTGGTTAGGGTGGCTCTGGTGGCCACCCTGGGGTGTTGTGTTTTCCTCACAAGGATCAGGAGGTATGATGGCATTTCCTAG

# Supplementary Figures and Tables

For more information on Supplementary Material and for details on the different file types accepted, please see [here](https://www.frontiersin.org/guidelines/author-guidelines#supplementary-material).

## Supplementary Figures

**
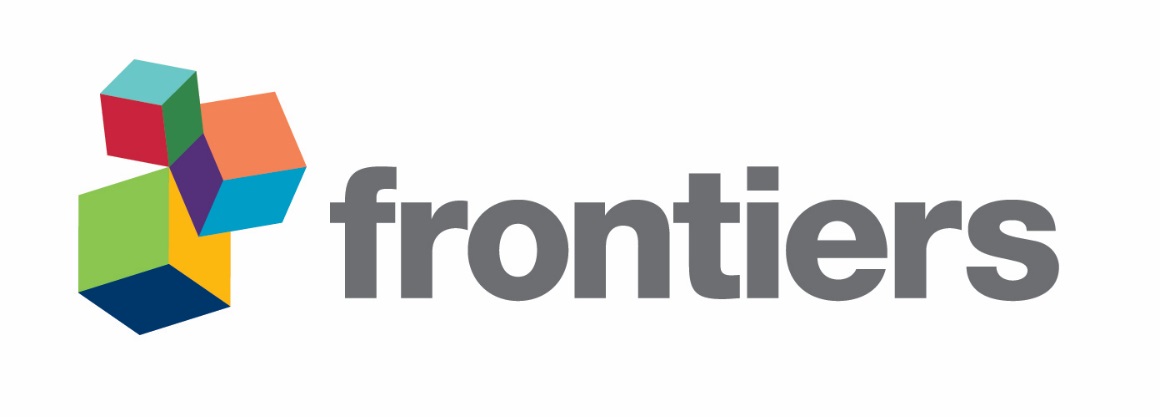
**

**Supplementary Figure 1.** The figure legends are required to have the same font as the main text, 12 point normal Times New Roman, single spaced. Please use a single paragraph for each legend and prepare the figures keeping in mind the PDF layout.
