# Supplementary figures and images for: The significance of CEACAM60, a carcinoembryonic antigen (CEA) homolog, as a tumor antigen in the porcine cancer model
Source: Front Immunol. 2026 May 13;17:1813834. doi: 10.3389/fimmu.2026.1813834 (PMC13212111; doi:10.3389/fimmu.2026.1813834)

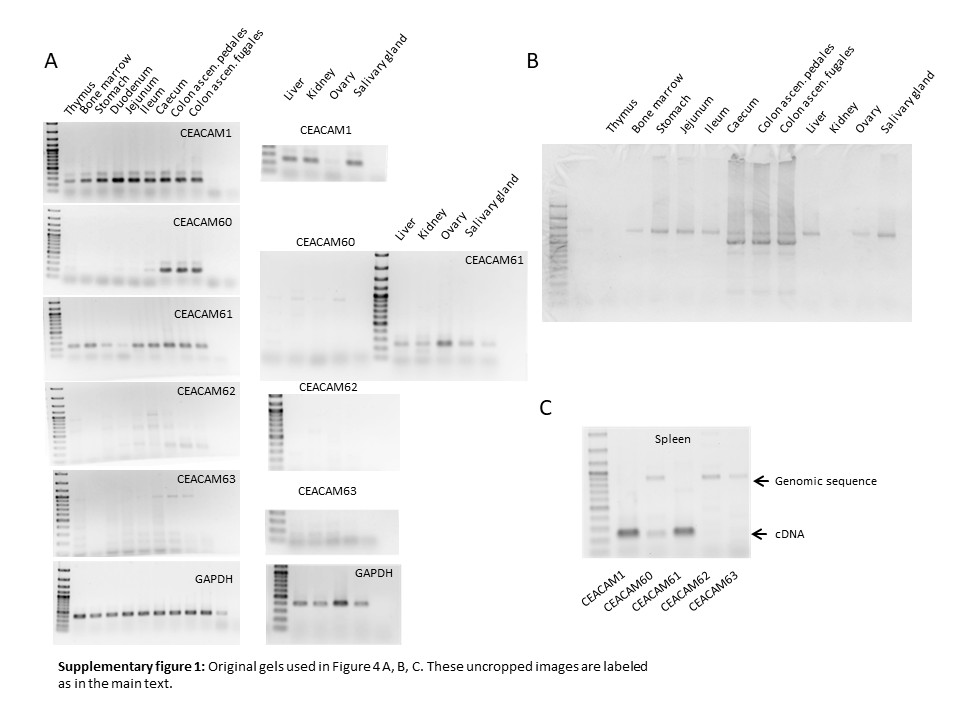

Supplement: Supplementary file 2 [file Image1.jpeg]
